# Tumour kinome re-wiring governs resistance to palbociclib in oestrogen receptor positive breast cancers, highlighting new therapeutic modalities

**DOI:** 10.1038/s41388-020-1284-6

**Published:** 2020-04-19

**Authors:** Sunil Pancholi, Ricardo Ribas, Nikiana Simigdala, Eugene Schuster, Joanna Nikitorowicz-Buniak, Anna Ressa, Qiong Gao, Mariana Ferreira Leal, Amandeep Bhamra, Allan Thornhill, Ludivine Morisset, Elodie Montaudon, Laura Sourd, Martin Fitzpatrick, Maarten Altelaar, Stephen R. Johnston, Elisabetta Marangoni, Mitch Dowsett, Lesley-Ann Martin

**Affiliations:** 1 Breast Cancer Now Toby Robins Research Centre, Institute of Cancer Research, London, SW7 3RP UK; 2 Biomolecular Mass Spectrometry and Proteomics, Bijvoet Center for Biomolecular Research and Utrecht Institute for Pharmaceutical Sciences, Utrecht University, 3584 CH Utrecht, The Netherlands; 3 CRUK, Bioinformatic Cofacility, Institute of Cancer Research, Sutton, SM2 5NG UK; 4 Proteomic Unit, Institute of Cancer Research, London, SW7 3RP UK; 5 Centre for Cancer Imaging, Institute of Cancer Research, Sutton, SM2 5NG UK; 6 Department of Translational Research, Institut Curie, Paris, France; 7 Breast Unit, Royal Marsden Hospital, London, SW3 6JJ UK; 8 Ralph Lauren Centre for Breast Cancer Research, Royal Marsden Hospital, London, SW3 6JJ UK

**Keywords:** Breast cancer, Cancer models

## Abstract

Combination of CDK4/6 inhibitors and endocrine therapy improves clinical outcome in advanced oestrogen receptor (ER)-positive breast cancer, however relapse is inevitable. Here, we show in model systems that other than loss of *RB1* few gene-copy number (CN) alterations are associated with irreversible-resistance to endocrine therapy and subsequent secondary resistance to palbociclib. Resistance to palbociclib occurred as a result of tumour cell re-wiring leading to increased expression of *EGFR, MAPK, CDK4, CDK2, CDK7, CCNE1* and *CCNE2*. Resistance altered the ER genome wide-binding pattern, leading to decreased expression of ‘classical’ oestrogen-regulated genes and was accompanied by reduced sensitivity to fulvestrant and tamoxifen. Persistent CDK4 blockade decreased phosphorylation of tuberous sclerosis complex 2 (TSC2) enhancing EGFR signalling, leading to the re-wiring of ER. Kinome-knockdown confirmed dependency on ERBB-signalling and G2/M–checkpoint proteins such as WEE1, together with the cell cycle master regulator, CDK7. Noteworthy, sensitivity to CDK7 inhibition was associated with loss of ER and *RB1* CN. Overall, we show that resistance to CDK4/6 inhibitors is dependent on kinase re-wiring and the redeployment of signalling cascades previously associated with endocrine resistance and highlights new therapeutic networks that can be exploited upon relapse after CDK4/6 inhibition.

## Introduction

The majority of breast tumours at primary diagnosis are oestrogen receptor alpha positive (ER). This knowledge has been exploited clinically by the development of endocrine therapies, which seek to deprive the hormone dependent tumour cells of oestrogen (E) using aromatase inhibitors (AI), or the use of antiestrogens such as tamoxifen or fulvestrant (Fulv) both of which compete with E for the ER. Antagonising ER leads to inhibition of cyclin dependent kinase (CDK)/cyclin activity and the maintenance of retinoblastoma protein (RB) in a hypophosphorylated state suppressing transcription of E2F-regulated genes and inhibiting progression through S-phase.

Unfortunately, the beneficial actions of existing endocrine treatments are attenuated by the ability of tumours to circumvent the need for steroid hormones, whilst in most cases, retaining the nuclear steroid receptors (reviewed by [1]). In this setting, aberrant growth factor signalling leading to altered expression of key downstream pathways, such as PI3K/AKT/mTOR and ERK1/2, converge at the level of cyclin D forcing cell cycle progression. Furthermore, ERK1/2 is also known to regulate AP1 (Fos/Jun) complexes, which in turn can drive transcription of *CCND1* [2]. In addition, deregulation of specific cell cycle components such as RB and p27^kip1^ can reduce the efficacy of ER inhibition [3]. Amplification of *CCND1* occurs in ~15% of breast cancer (BC) [4] and overexpression in a larger proportion [5] has been associated with resistance to endocrine therapy [6–8]. This high degree of heterogeneity in adaptive mechanisms during the course of BC progression highlights the importance of finding common nodes responsible for to therapeutic failure.

As proliferation is a hallmark of endocrine resistant tumours, targeting cell cycle regulation has provided an attractive proposition. Indeed, recent studies suggest many cancer cells might be addicted to high CDK4/6 activity [9]. A number of CDK inhibitors have been developed but the most widely used to date is palbociclib (PD-0332991), an orally available selective inhibitor of CDK4 and CDK6 kinases, which is capable of blocking RB-phosphorylation resulting in G1 arrest [10].

The combination of CDK4/6 inhibitors and endocrine therapy have been shown to improve clinical outcome in patients with ER+ metastatic BC [11–13], and have since become approved as 1st and 2nd line treatment options. However, not all patients will benefit from such combination therapy and many will eventually relapse with acquired resistance to combined treatment through poorly characterised mechanisms. In order to address this, we generated models of acquired resistance to palbociclib and showed that other than copy number (CN) loss of *RB1*, few genetic changes are associated with resistance to CDK4/6 inhibition. Upregulation of *CCNE1* and *CCNE2* was evident in all resistant models. We provide evidence that prolonged CDK4 blockade enhances EGFR/ERBB signalling, as a result of reduced TSC2-phosphorylation, which impacts downstream on ER signalling leading to an altered ER-cistrome and reduced sensitivity to subsequent endocrine blockade. Overall, we show that resistance to CDK4/6 inhibitors is dependent on kinase re-wiring and the redeployment of signalling cascades previously associated with endocrine resistance. Our study highlights the potential clinical utility of targeting the ERBB-signalling axis or cell cycle via perturbation of CDK7 (cell cycle master regulator) or WEE1 (G2/M Checkpoint), according to the mode of resistance acquired to long-term CDK4/6 treatment.

## Material and methods

Detailed materials and methodology is provided in the Supplementary STAR file.

### Cell culture

Human BC cell lines MCF7, T47D, HCC1428, ZR75.1 and SUM44 were purchased from ATCC and Asterand. Cells were cultured in phenol red free RPMI containing 10% foetal bovine serum (FBS) and 1 nM estradiol (E2). LTED derivatives were cultured in phenol red free RPMI supplemented with 10% dextran charcoal stripped (DCC) FBS, as previously described [14]. Palbociclib-resistant cell lines were generated by long-term culture of parental cell lines in the continuous presence of 1 µM palbociclib until resistance developed (in average 5–6 months for all the cell lines). Resistance was authenticated by lack of response to escalating concentrations of palbociclib in comparison with their wild-type progenitor cell line and routine passage in the presence of the drug. From that point, palbociclib-resistant cell lines were routinely cultured in the presence of 1 µM palbociclib. Palbociclib was removed from the media 48 h prior to each experiment unless otherwise stated. All cell lines were authenticated by short tandem repeats (STR) profiling and routinely screened for mycoplasma contamination.

### Proliferation and spheroid assays

Cell viability were carried as detailed previously [14]. In brief, parental cell lines were cultured in DCC medium for 3 day. Cells were seeded into 96 well plates. The following day monolayers were treated with the drug concentrations indicated for 6 days with a medium change on day 3. Viability was measured using TiterGlo according to the manufacturers’ instructions (Promega, UK). Statistical analysis for the drug studies has been performed using Wilcoxon Matched pairs test using Graph-Prism.

Spheroid cultures were generated by seeding 2500 cells per well of a 96 well ultra-low-attachment plate (Costar). Plates were spun at 900 × *g*_ave_ for 10 min. Spheres were formed over 72 h and subsequently treated with the drugs as indicated for 7 days. Proliferation was assessed using Celigo S (Nexcelom Bioscience).

### Immunoblotting

Immunoblotting was carried out, as previously described [14]. In brief whole cell lysates were resolved using SDS-PAGE followed by western blotting. Membranes were washed, blocked using 5% milk powder then immunoprobed with antibodies directed against the relevant proteins.

### Real-time quantitative PCR

Taqman gene expression assays (Applied Biosystems) were used to quantify *TFF1* (Hs00907239_m1), *CCND1* (Hs00765553_m1), *PDZK1* (Hs00275727_m1), *RB1* (Hs01078066_m1), *EGFR* (HS01076090_m1), *MAPK1* (Hs01046830_m1), *MAP3K1* (Hs00394890_m1), *CDK2* (Hs01548894_m1), *CDK4* (Hs00364847_m1), *CDK7* (Hs00361486_m1), *CCNE1* (Hs01026536_m1), *CCNE2* (Hs00180319_m1) together with *FKBP15* (Hs00391480_m1) as housekeeping gene to normalise the data. The relative quantity was determined using ΔΔCt, according to the manufacturer’s instructions (Applied Biosystems).

### Gene expression microarray analysis of cell lines

Global gene expression analysis was carried out for MCF7, MCF7 LTED and T47D human BC cell lines along with their palbociclib-resistant derivatives (GSE98987). Data was normalised and handled, as previously described [14]. Gene set enrichment analysis was carried out using the GSEA v:2.0.13 GSEA pre-ranked tool (http://software.broadinstitute.org/GSEA/msigdb/annotate.jsp and G-profiler).

### Gene copy number analysis

DNA was extracted from the target cell lines using QIAamp DNA mini kit, according to the manufacturer’s instructions (Qiagen). Exome libraries were generated, as previously described [15]. Data was deposited in the sequence read archive: BioProject (PRJNA604231).

### ChIP-seq

ChIP-seq was performed, as previously described [14, 16] using ER antibody (HC-20 (sc-543 Santa Cruz)) or IgG (Dako). Statistical tests and cut-offs were selected based on published recommendations [17].

### Dimethyl labelling and phosphoproteomics

Dimethyl labelling was carried out, as reported previously [18]. Samples were run through LC-MS/MS using LTQ Velos Orbitrap MS (Thermo Scientific). Raw data were processed using MaxQuant 1.5.1.0. Ti4+-IMAC phosphopeptide enrichment was performed, as previously described [19]. LC-MS/MS measurements were performed, by coupling an Agilent 1290 Infinity II LC system to a QExactive Plus mass spectrometer (Thermo Scientific). Phosphoproteomic raw spectra were processed with MaxQuant (version 1.5.2.8). Quantified phosphodata were processed using PaDua [20], a custom Python in house-package and network reconstruction and pathway identification were conducted using Photon [21] and PhosphoPath [22], respectively. Phosphoproteomics [PRIDE PXD005514] and Proteomics data [PRIDE PXD005611] have been deposited in the ProteomeXchange.

### Human tumour xenografts modelling relapse on AI therapy

In vivo studies were carried out in ovariectomized 8- to 12-week-old female BALB/c- nude mice in accordance with Home Office guidelines and approved by the Institute of Cancer Research Ethics Committee. Xenografts modelling patients resistant to AI (MCF7 LTED) were treated with 100 mg/kg palbociclib administered daily by oral gavage or vehicle control. The study operator was blinded to the treatments. Overall statistical differences were calculated using the Wilcoxon signed-rank test if the variance was not equal and failed the normality test otherwise paired *t*-tests were used.

### In vivo PDX efficacy studies

Palbociclib resistant PDX (see star file) were generated and transplanted into female 8-week-old Swiss nude mice in accordance with institutional guidelines and the rules of the French Ethics Committee (project authorisation no. 02163.02). Mice were randomly assigned to the control or treated groups (4–7 mice per group). Palbociclib was administered orally twice per week at 50 mg/kg. Everolimus was administered orally at a dose of 2.5 mg/kg, 3 days per week. Fulv was administered by intramuscular injection at a dose of 50 mg/kg once a week. AZD1775 and neratinib were administered orally at 90 mg/kg and 40 mg/kg, 5 days per week, respectively. Treatments were administered for 60 days. The statistical significance was determined by unpaired *t*-test.

### RNAi kinome library screen

MCF7^PalboR^, MCF7 LTED^PalboR^, T47D^PalboR^ and T47D LTED^PalboR^ cell lines were transfected using the ON-TARGETplus siRNA Library-Human Tyrosine Kinases (Dharmacon) targeting 709 protein kinases. After 6 days, cell viability was assessed using CellTiterGlo assay (Promega). A library screen for each cell line was performed in duplicate and repeated 2–3 times. The dynamics of each library screen was assessed by calculating *Z* prime values. The threshold of acceptance was set as *Z*′ > 0.3 [23].

## Results

### Effect of palbociclib on cell growth of endocrine-sensitive and long-term oestrogen-deprived BC cell lines

A panel of endocrine-sensitive and long-term oestrogen deprived (LTED) BC cell lines modelling relapse on an AI, were evaluated for their sensitivity to the CDK4/6 inhibitor palbociclib: MCF7, SUM44, T47D, ZR75.1 and HCC1428 cell lines along with their LTED derivatives were assessed both in the presence and absence of 17β−estradiol (E2).

The cell lines showed varying degrees of sensitivity to palbociclib (Supplementary Fig. S1a). MCF7, SUM44, T47D, ZR75.1 and HCC1428 showed a dose-dependent decrease in proliferation in the presence of E2 with GI_50_ values ranging between 100 and 300 nM. In the absence of E2, modelling the effect of an AI, proliferation dropped significantly in all cell lines and the addition of palbociclib at concentrations between 500 and 1000 nM showed a further reduction of ~50%. This suggested that in the absence of E2, palbociclib targeted those cells which had partial resistance to E2 deprivation.

LTED cell line derivatives showed sensitivity to escalating concentrations of palbociclib in the absence and presence of E2 with GI_50_ values ranging between 100 and 700 nM (Supplementary Fig. S1a). Furthermore, the antiproliferative effect was confirmed in MCF7 LTED spheroids (Supplementary Fig. S1b). The combination of palbociclib with either 4-hydroxytamoxifen (4OHT) or Fulv showed enhanced antiproliferative activity compared with either agent alone (Supplementary Fig. S1c). Immunoblot analysis of MCF7 LTED cells treated with palbociclib alone or in combination with 4OHT or Fulv (Supplementary Fig. S1d), showed a decrease in CDK4/6 targets (phosphorylated and total RB, p107 and FOXM1) and increased expression of p130, matching previous reports suggesting that p130 is found predominantly in quiescent cells [24]. The combination of CDK4/6 inhibition with endocrine therapy further reduced phosphorylation of RB and p107. Assessment of downstream signalling targets of ER showed a slight but noticeable accumulation of cyclin D1 and cyclin D2, but decreased abundance of cyclin D3. A marked increase in cyclin E1 upon palbociclib treatment, was evident. The CDK inhibitor p21 increased with palbociclib treatment demonstrating accumulation following cell cycle inhibition.

Taken together, these data indicate palbociclib markedly enhances inhibition of RB phosphorylation by anti-oestrogens and E deprivation, reducing key signalling mediators and targets involved in cell proliferation.

### Resistance to CDK4/6 inhibition results via multiple mechanisms

**Fig. 1:**
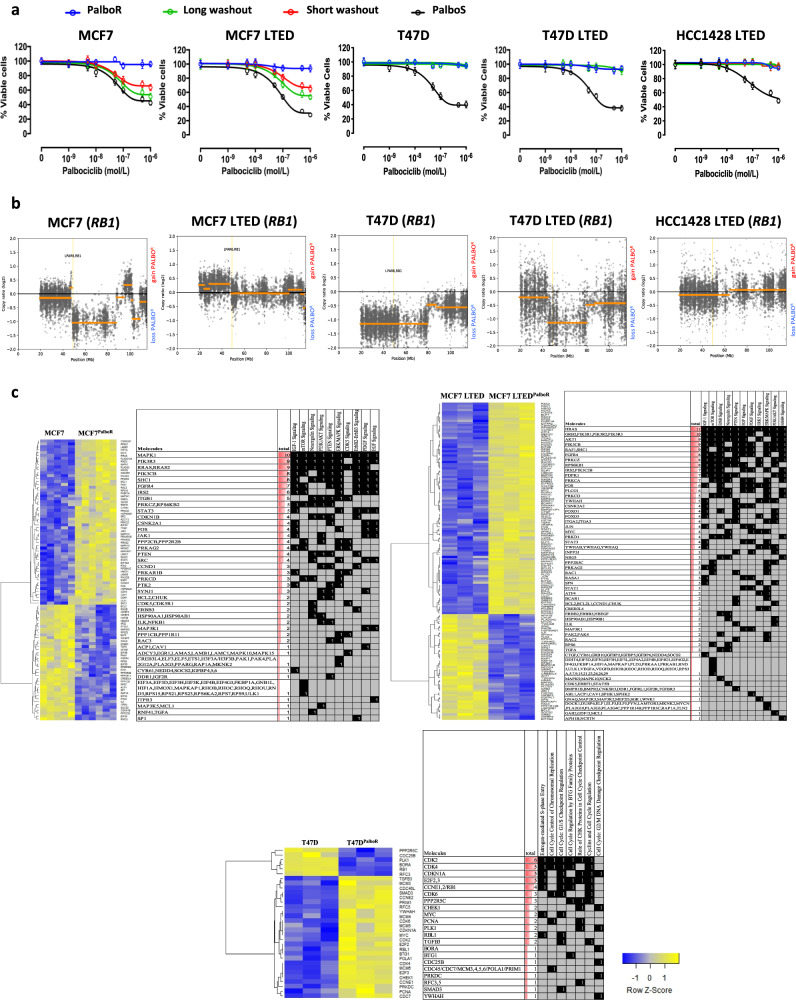
Generation and characterisation of palbociclib resistance models. **a** Antiproliferative effect of palbociclib in several palbociclib-resistant (PalboR, blue lines) and -sensitive (PalboS, black lines) cell lines. For washout experiments, palbociclib was omitted from the growth medium for a period of 2 weeks (short, red line) and 4 weeks (long, green line) and then re-challenged with escalating concentrations of palbociclib (*n* = 3 biological and *n* = 8 technical replicates). Data represents % viable cells compared with vehicle control for each cell line. Error bars represent mean ± SEM. **b** Copy number variation comparing palbociclib resistance versus sensitivity in MCF7, MCF7 LTED, T47D, T47D LTED and HCC1428 LTED cell lines. **c** Identification of pathways and genes associated with resistance to palbociclib in MCF7, MCF7 LTED and T47D cell models (*n* = 3 biological replicates).

In order to identify the pathways associated with resistance to palbociclib, we treated a panel of cell lines with varying phenotypic backgrounds (MCF7, MCF7 LTED, T47D, T47D LTED, SUM44, SUM44 LTED, HCC1428 and HCC1428 LTED) long-term in the presence of palbociclib (1μM). Of the 8 cell lines, 5 palbociclib-resistant models were successfully generated, three of which, were also resistant to E-deprivation (MCF7^PalboR^, MCF7 LTED^PalboR^, T47D^PalboR^, T47D LTED^PalboR^ and HCC1428 LTED^PalboR^). Resistance was authenticated by culturing the resistant cell lines with escalating concentrations of palbociclib in comparison with their wild-type progenitor (Fig. 1a). Furthermore, assessment of key cyclins and CDKs required for S, G2 and M phase entry were evaluated in MCF7 LTED^PalboR^ and shown to be elevated compared with the drug-treated parental cell line (Supplementary Fig. S2a).

In order to test the stability of the resistant phenotype, cell lines were cultured in the absence of palbociclib for up to 4 weeks (Fig. 1a). Washout of MCF7^PalboR^ and MCF7 LTED^PalboR^ derivatives partially re-sensitised them to the antiproliferative effect of palbociclib, an observation confirmed in vivo (Supplementary Fig. S2b) suggesting the phenotype was plastic. In contrast, the T47D^PalboR^, T47D LTED^PalboR^ and HCC1428 LTED^PalboR^ cell lines remained resistant.

To identify molecular features associated with palbociclib resistance, we carried out assessments of gene copy number (CN) alterations using exome sequencing (Fig. 1b). Initially, we focussed on genes associated with cell cycle. Similar to our previous finding [25], T47D^PalboR^ and T47D LTED^PalboR^ both showed CN loss of one of the two *RB1* alleles and an acquired mutation at the splice site of the second allele in T47D^PalboR^, and an acquired frameshift deletion in the second allele in T47D LTED^PalboR^. These alterations were accompanied with reduced transcript and protein abundance, the latter remaining reduced after drug washout (Supplementary Fig. S2c, d). In contrast, HCC1428 LTED^PalboR^ cells showed no change in *RB1* CN, but loss of both mRNA transcript and protein. As yet, the mechanism by which this occurs is unclear. MCF7 LTED^PalboR^ showed no *RB1* CN change and expression at both the transcript and protein level was similar to the parental cell line. MCF7^PalboR^ showed partial CN loss of *RB1* when taking the triploid status of chromosome 13 into consideration [26] and a concordant reduction in both *RB1* transcript and protein. Of note, upon drug washout, RB increased although not to the level seen within the parental cell line. This increase in RB may account for the partial re-sensitisation to palbociclib in this model (Fig. 1a and Supplementary Fig. S2d). No other genetic changes that could be associated with resistance to palbociclib could be identified.

To characterise the resistant phenotype further, we assessed alterations in global gene expression by comparing the parental and palbociclib-resistant MCF7 isogenic cell lines together with T47D^PalboR^, as a comparator based on their overall *RB1* CN loss. As expected, pathway analysis (Fig. 1c and Supplementary File S1) showed that the T47D^PalboR^ model was dominated by cell cycle pathways and enriched for genes such as *CDK2, CCNE1* and *CHEK1* associated with the G1/S, S-phase entry and chromosomal replication. The MCF7 LTED^PalboR^ cells displayed decreased expression of proliferation-associated pathways, irrespective of their resistant phenotype, and concordant with their slower proliferation rate (Supplementary Fig. S3a, b). Pathway analysis showed that resistance in both MCF7^PalboR^ and MCF7 LTED^PalboR^ cells was associated with altered growth factor signalling such as IGF1, PDGF, ERBB2-ERBB3, ERBB4, neuregulin, EGF and ERK/MAPK. Common genes within these pathways included *HRAS, MAPK1* and those encoding subunits of PI3K (Fig. 1c). Further interrogation using gene set enrichment analysis (GSEA) showed a reduction in genes negatively regulating EGFR and ERBB signalling and an increase in those associated with enhanced activity (Supplementary Fig. S3c). In order to further validate this observation, we conducted RT-qPCR of target genes and showed that expression was concordant with the global gene analysis (Supplemental Fig. S4).

### Phosphoproteomic analysis identifies changes in cell cycle and growth factor signalling pathways associate with resistance to palbociclib

In order to investigate changes in global protein abundance and phosphorylation associated with the palbociclib-resistant phenotype, we used mass-spectrometry-based approaches. Comparison of dimethyl labelling of MCF7 and MCF7^PalboR^ cells showed increased abundance of cyclin D3, cyclin D1, cyclin E1 and CDK4 largely concordant with our gene expression data. MCF7 LTED^PalboR^ cells showed increased abundance of cyclin D1 and CDK5 (Supplementary File S2). Immunoblot analysis confirmed that all palbociclib-resistant cell lines showed increased abundance of cyclin E1 despite the absence of CN alteration, in contrast to our previous findings [25].

**Fig. 2:**
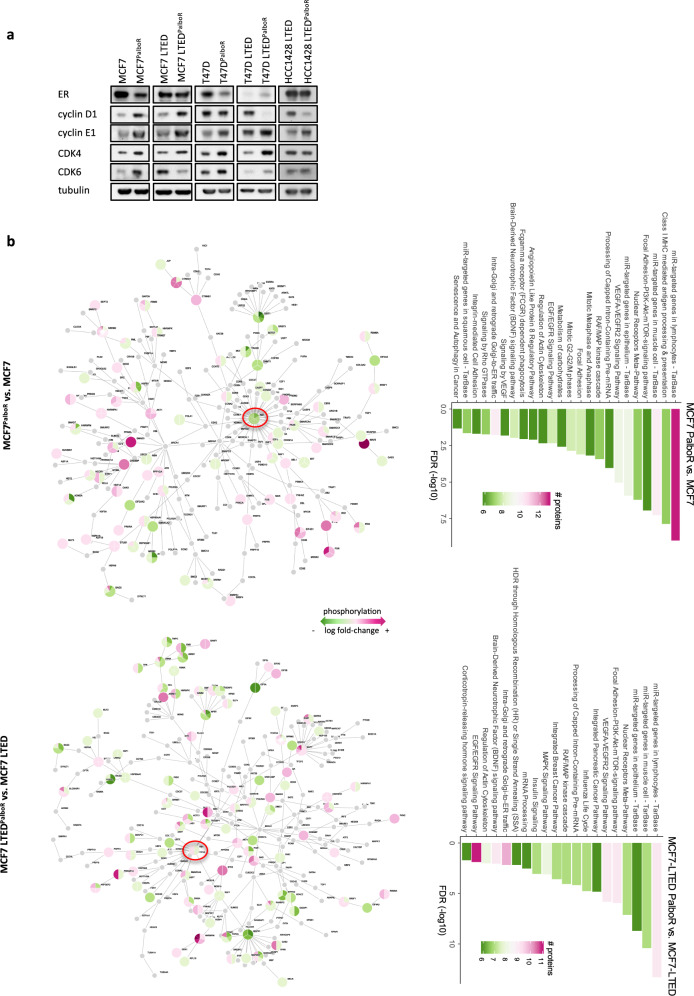
Assessment of global changes in phosphorylated proteins upon palbociclib resistance. **a** Abundance of ER and cell cycle markers in palbociclib-sensitive and -resistant in MCF7, MCF7 LTED, T47D, T47D LTED and HCC1428 LTED cell lines (*n* = 3 biological replicates). **b** Schematic representation of phosphorylation networks using Photon comparing parent and palbociclib-resistant (PalboR) derivatives followed by identification of pathways associated with resistance using PhosphoPath (*n* = 3 biological replicates). RB1 marked with red circle.

Both HCC1428 LTED^PalboR^ and T47D LTED^PalboR^ showed a decrease in cyclin D1 abundance compared with the other cell lines. CDK4 was elevated in all palbociclib-resistant cell lines with the exception of the HCC1428 LTED^PalboR^ (Fig. 2a). Assessment of ER levels showed no change in *ESR1* transcript in MCF7 versus MCF7^PalboR^ but a slight decrease in protein abundance was evident in both the dimethyl labelling and immunoblot (Fig. 2a, Supplementary Fig. S5a and Supplementary File S2). MCF7 LTED^PalboR^ showed a slight increase in *ESR1* transcript, although this was not reflected at the protein level. HCC1428 LTED^PalboR^ and T47D^PalboR^ models showed a similar reduction in *ESR1* transcript. This was reflected as a decrease in protein abundance in the T47D^PalboR^ but not HCC1428 LTED^PalboR^. As expected, T47D LTED and palbociclib-resistant derivative showed negligible ESR1 transcript or protein.

To identify differential phosphorylation events in the MCF7^PalboR^ and MCF7 LTED^PalboR^ derivatives compared with their parental cell lines, we used two visualisation tools Phosphopath [22] and Photon [21]. These revealed pathways associated with cell cycle progression, EGFR, MAPK and PI3K/AKT/mTOR, concordant with the gene networks identified previously in these resistant cell lines (Fig. 1c, Fig. 2b and Supplementary File S2). RB was shown to be similarly phosphorylated in MCF7 LTED^PalboR^ compared with their parental cell line. In contrast, the MCF7^PalboR^ showed a reduction in both phosphorylation and abundance in keeping with the CN loss identified in our exome analysis (Fig. 1b). In order to validate some of the key observations, related to ERBB signalling, immunoblot analysis was carried out and shown to be concordant (Supplementary Fig. S5b). Taken together, these data suggest tumour rewiring leading to increased ERBB/MAPK signalling influences sensitivity to CDK4/6 inhibition.

### Resistance to palbociclib associates with downregulation of ER-classical activity ChIP-seq

**Fig. 3:**
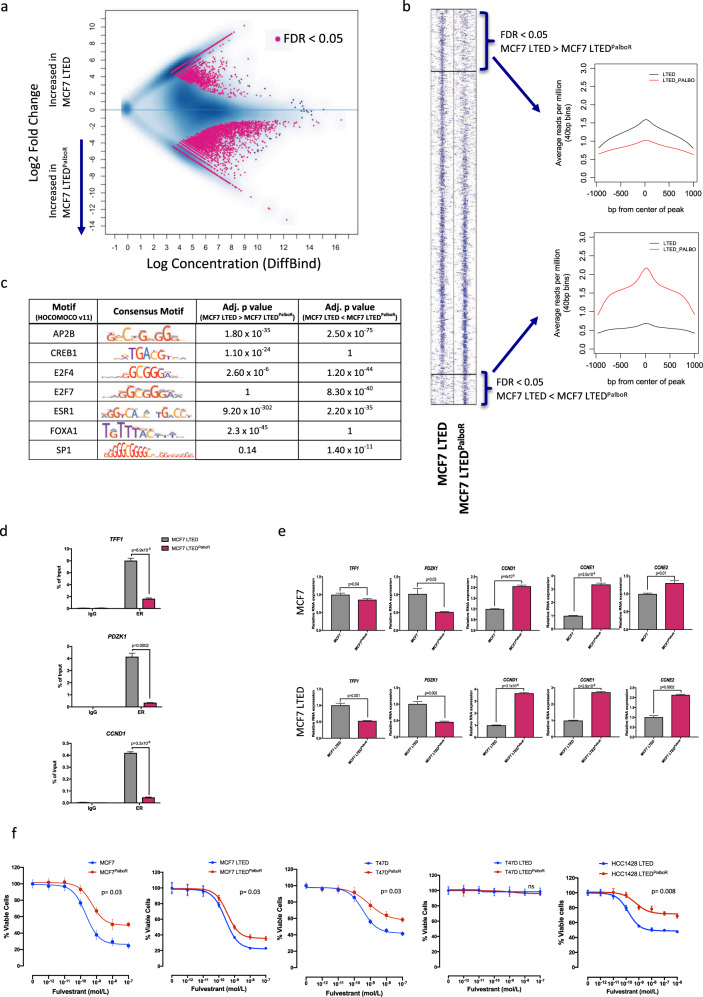
Resistance to palbociclib associates with downregulation of ER-classical activity assessed by ChIP-seq. **a** MA plot showing the differential binding affinity of ER. The *x*-axis shows log concentration of sequenced tags per peak; *y*-axis represents log2 fold change of MCF7 LTED/ MCF7 LTED^PalboR^ (*n* = 3 biological replicates). **b** Heatmap depicting binding peak intensities, which are common or different between the two cell lines. The window represents ±1 kb regions from the centre of the binding event. **c** Motif analysis of common and augmented ESR1 peaks from MCF7 LTED versus MCF7 LTED^PalboR^. **d** Effect of palbociclib resistance in recruitment of the ER to *TFF1*, *PDZK1* and *CCND1* promoters. Error bars represent means ± SEM. (*n* = 2 biological replicates). **e** Effect of palbociclib resistance on expression of *TFF1*, *PDZK1*, *CCND1, CCNE1* and *CCNE2* in MCF7 and MCF7 LTED cell lines. Error bars represent means ± SEM. **f** Effect of escalating concentrations of fulvestrant on the proliferation of MCF7, MCF7 LTED, T47D, T47D LTED and HCC1428 LTED and their corresponding palbociclib (PalboR) resistant cell lines. Data represents % viable cells compared with vehicle control for each cell line. Error bars represent mean ± SEM.

As previous studies have provided evidence that increased ERBB activity can alter the ER-genomic binding pattern [27], we carried out comparative ChIP-seq analysis of ER in both the parental MCF7 LTED and MCF7 LTED^PalboR^ cells. Whilst the majority of ER-binding sites were similar between the palbociclib-sensitive and -resistant MCF7 LTED (Fig. 3a, b) some differences were apparent. For instance, motif analysis showed enrichment of ESR1, FOXA1 and CREB1 binding sites in the MCF7 LTED compared with the resistant cell line. In contrast, the MCF7 LTED^PalboR^ cells showed enrichment of AP2B, SP1, E2F7 and E2F4 motifs (Fig. 3c). Further analysis of the gene expression data showed an increase in early E response signalling and a decrease in late E response signalling in the MCF7 LTED^PalboR^ (Supplementary Fig. S6a). A core set of genes associated with both early and late response were interrogated further. Strikingly, ER-regulated genes associated with cell cycle were downregulated in the palbociclib-resistant cells and there was a shift towards dependence on SP1 transcription factors (Supplementary Fig. S6b, c). This suggested a trend towards loss of ER binding at ‘classically’ E-regulated genes, which was confirmed by ChIP-qPCR analysis of ER-recruitment to the promoter regions of the *TFF1*, *PDZK1* and *CCND1* in the palbociclib-resistant cell line (Fig. 3d). This was concordant with decreased expression of *TFF1* and *PDZK1*. However, the loss of ER-binding at the *CCND1* promoter had no impact on *CCND1* expression in the palbociclib-resistant cells suggesting its increased expression, together with both *CCNE1* and *CCNE2*, resulted from alternate mitogenic cues (Fig. 3e). This reduction in expression of classical E-regulated genes was recapitulated in MCF7^PalboR^ cell line. Assessment of the antiproliferative effect of Fulv and 4OHT revealed a significant reduction in sensitivity in all palbociclib-resistant cell lines harbouring ER compared with their respective parental cell lines and was most pronounced in those with decreased or lost *RB1* expression (Fig. 3f and Supplementary Fig. S7a). Nonetheless, these data suggest that whilst ER remains important in the resistant phenotype, its transcriptional programme may be altered as a result of chromatin remodelling in response to prolonged exposure to CDK4/6 blockade.

### Increased ERBB signalling results from sustained CDK4/6 blockade

**Fig. 4:**
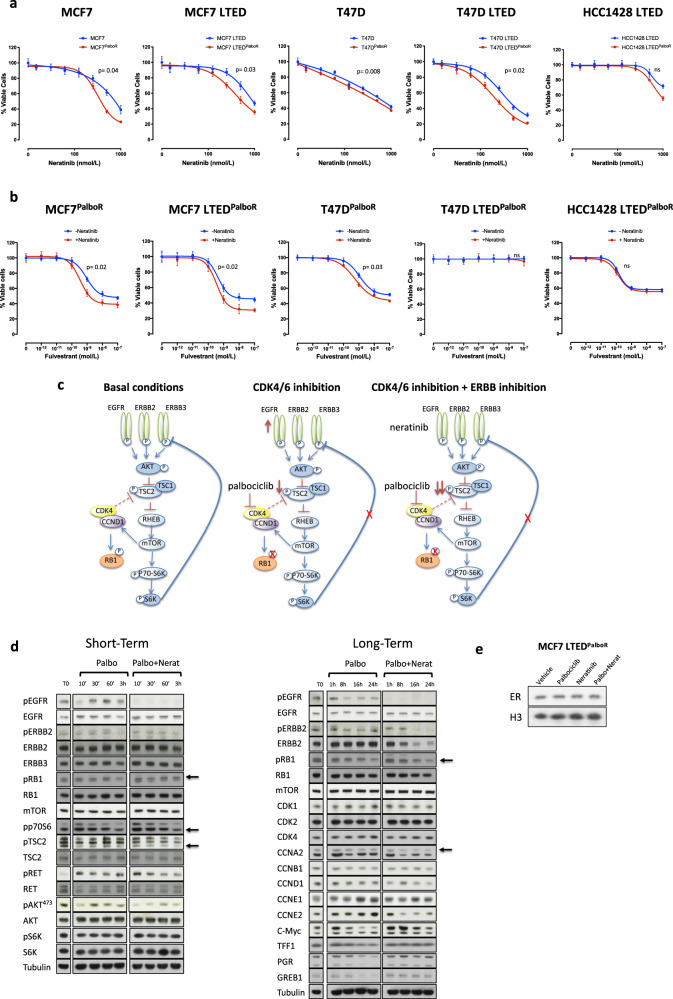
Increased ERBB signalling following sustained palbociclib resistance. **a** Antiproliferative effect of neratinib in MCF7, MCF7 LTED, T47D, T47D LTED and HCC1428 LTED and their corresponding palbociclib-resistant (PalboR) derivatives. Data represents % viable cells compared with vehicle control for each cell line. Error bars represent mean ± SEM. **b** Effect of escalating concentrations of fulvestrant both in the presence or absence of neratinib (500 nM) in MCF7, MCF7 LTED, T47D, T47D LTED and HCC1428 LTED and their corresponding palbociclib-resistant (PalboR) cell lines. Data represents % viable cells. Error bars represent mean ± SEM. **c** Schematic representation of the effect of CDK4/6 inhibition and cross-talk with receptor tyrosine kinase signalling pathway. **d** Immunoblotting showing expression levels of several cell cycle, growth factor signalling and ER-regulated markers in MCF7 LTED^PalboR^ cell lines following single treatment with palbociclib (1 μM) or in combination with neratinib (500 nM) over a time course of 24 h. **e** Immunobloting showing abundance of nuclear ER in MCF7 LTED^PalboR^ cell lines after treatment with palbociclib (1 μM), neratinib (500 nM) or the combination of both.

As increased EGFR and ERBB2 were evident in the MCF7 isogenic palbociclib-resistant cell lines, we assessed sensitivity to the anti-proliferative effect of the pan-ERBB inhibitor neratinib. All cell lines showed a significant reduction in proliferation in response to the drug. Noteworthy, with the exception of T47D^PalboR^ and HCC1428 LTED^PalboR^, all palbociclib-resistant cell lines showed enhanced sensitivity to neratinib compared with their respective parental model (Fig. 4a). This observation was confirmed with a second EGFR/ERBB inhibitor, AZD8931 (Supplementary Fig. S7b). As EGFR and ERBB2 are known to alter ER-function, we hypothesised that perturbation of these growth factors may re-sensitise the palbociclib-resistant cell lines to Fulv (Fig. 4b). Indeed, a shift in sensitivity in response to Fulv was evident in the presence of neratinib in the MCF7 isogenic models and to a lesser degree in the T47D^PalboR^ cell line but not the HCC1428 LTED^PalboR^. As expected, the T47D LTED^PalboR^, which no longer expresses ER, showed no response to Fulv.

### CDK4/6 blockade leads to increased EGFR/ERBB expression resulting in downregulation of ER signalling

Cross-talk between EGFR/ERBB, mTOR and CDK4 signalling is well documented [28–30]. We hypothesised that blockade of CDK4 leads to the reduction of phosphorylation of TSC2 together with a reduction in S6 kinase thereby negating the negative feedback loop between TSC2 and EGFR (Fig. 4c). To address this, we investigated the expression of these target proteins over a long and short time course (Fig. 4d and Supplementary File S3). MCF7 LTED^PalboR^ cells were serum-starved and treated with palbociclib alone or in combination with neratinib. Addition of palbociclib resulted in an increase in total EGFR abundance accompanied with an increase in phosphorylation after 30 min exposure to palbociclib. Phosphorylated RET showed a marked increase as early as 10 min post palbociclib treatment. Contrastingly, pTSC2 was significantly reduced at the earliest time point post addition of palbociclib compared with vehicle. As expected, the combination of palbociclib and neratinib reduced pTSC2 and both pEGFR and pERBB2, which remained ablated for the duration of the time course. Surprisingly, RET phosphorylation was also significantly reduced. Assessment of cyclin abundance showed maintenance of cyclin A2 in the presence of palbociclib demonstrating resistance to therapy. However, addition of neratinib reduced cyclin A2, cyclin D1 and cyclin E2. Most noteworthy, assessment of the ER-regulated gene products TFF1, PGR, GREB1 and c-MYC showed a reduction in the presence of palbociclib, which was rescued by the addition of neratinib. Further assessment of the impact of palbociclib on nuclear ER content showed no effect compared with vehicle control or the combination with neratinib suggesting nuclear shuttling was not the cause of reduced classical ER-activity (Fig. 4e). In summary, these data suggest that prolonged treatment with palbociclib induces tumour re-wiring leading to increased expression of growth factor signalling which provides an alternate mitogenic cue in RB competent models which is sensitive to EGFR/ERBB inhibition.

### Identification of kinases associated with resistance to CDK4/6 inhibitions

**Fig. 5:**
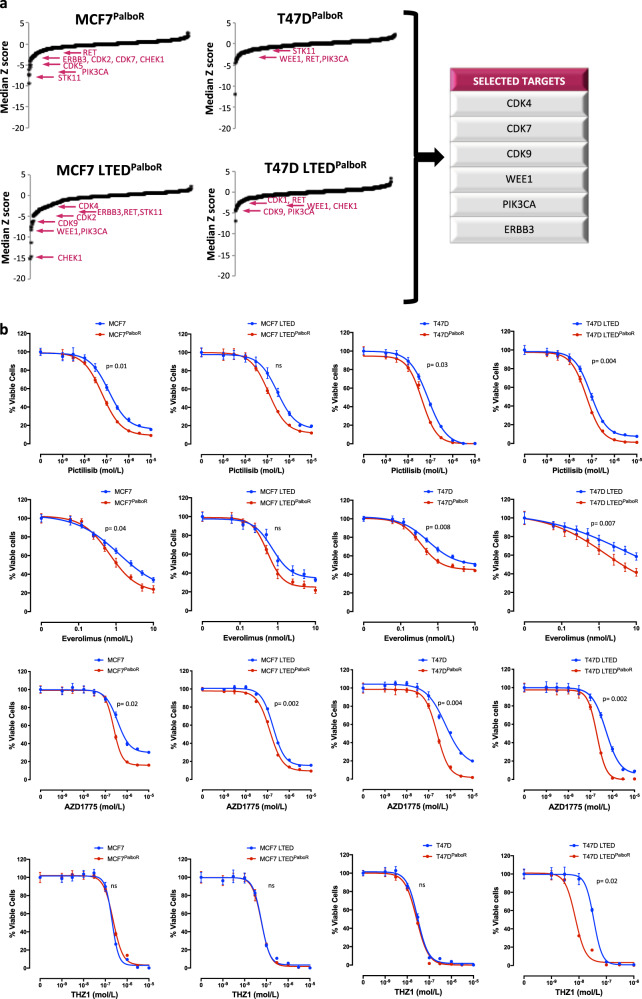
siRNA kinome knockdown identified several kinases associated with palbociclib resistance. **a** siKinome identified targets involved in palbociclib resistance (PalboR) (*n* = 2 biological and *n* = 3 technical replicates). **b** Effect of escalating doses of pictilisib (PI3K inhibitor), everolimus (mTORC1 inhibitor), AZD1775 (WEE1 inhibitor) and THZ1 (CDK7 inhibitor) in MCF7^PalboR^, MCF7 LTED^PalboR^, T47D^PalboR^ and T47D LTED^PalboR^ versus their corresponding parental cell lines (*n* = 3 biological and *n* = 8 technical replicates). Data represents % viable cells compared with vehicle control for each cell line. Error bars represent means ± SEM.

Based upon our previous findings, we hypothesised that elucidation of kinase re-wiring events may identify new therapeutic ‘Achilles heels’ associated with resistance to CDK4/6 inhibition. To address this, we assessed cell viability in response to a kinome knockdown screen (siRNA) targeting 709 kinases in the palbociclib-resistant cell lines (Fig. 5a and Supplementary File S4). T47D^PalboR^ and T47D LTED^PalboR^, which showed loss of RB function as their governing mode of resistance, were sensitive to loss of PIK3CA, RET and G2/M checkpoint regulator WEE1. MCF7^PalboR^ and MCF7 LTED^PalboR^ models showed susceptibility to loss of ERBB3 and dependency on multiple CDKs, which appeared cell line specific. Similar to the T47D, the MCF7 LTED^PalboR^ showed susceptibility to WEE1 loss. To address this further, proliferation assays targeting the key kinases identified were carried out in the parental and palbociclib-resistant cell lines (Fig. 5b). Perturbation of PI3K/mTOR signalling with pictilisib and everolimus, respectively, showed enhanced sensitivity in all palbociclib-resistant models compared with their corresponding parental cell lines. Inhibition of WEE1 with AZD1775 effectively suppressed proliferation in all cell lines tested, and similarly showed enhancement of sensitivity in the palbociclib-resistant cells compared with parental with the exception of MCF7 LTED^PalboR^ which was less pronounced. Noteworthy, whilst CDK7 was highlighted as a potential target, both parental and palbociclib-resistant cell lines appeared equally sensitive with the exception of T47D LTED^PalboR^ (*RB*-loss, ER loss), which showed almost a 10-fold shift in sensitivity. Inhibition of CDK9 had no significant impact in the palbociclib-resistant lines tested (Supplementary Fig. S7c).

Taken together, these data confirm our finding that growth factor receptor signalling associates with resistance to CDK4/6 inhibition and highlights the potential to target this, CDK7 or G2/M checkpoint proteins such as WEE1 upon progression on palbociclib.

### Effect of everolimus, neratinib and AZD1775 in PDX models of palbociclib resistance

**Fig. 6:**
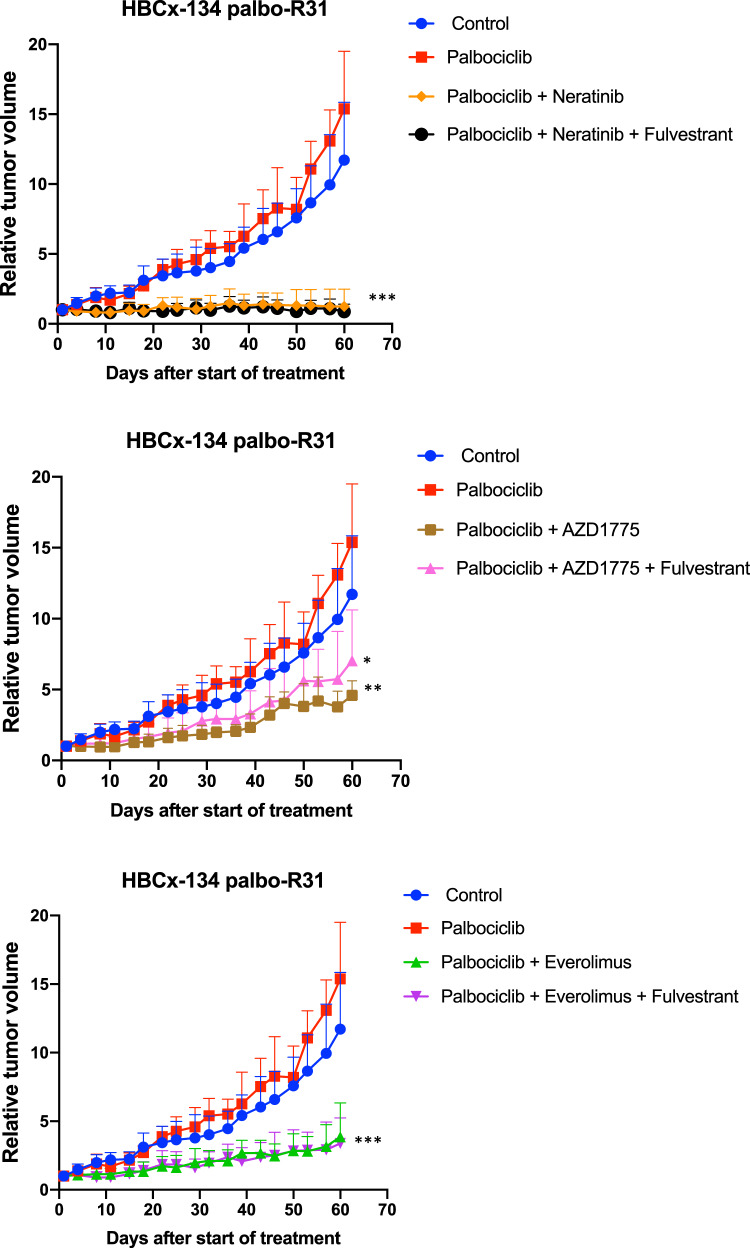
Effect of everolimus, neratinib and AZD1775 on tumour progression in palbociclib resistant PDX models. Long-term study assessing changes in relative tumour volumes over 60 days of treatment with palbociclib alone (50 mg/kg) or in combination with everomilus (2.5 mg/kg), neratinib (40 mg/kg) and AZD1775 (90 mg/kg) in the presence or absence of fulvestrant (50 mg/kg) in a PDX model of resistant to palbociclib (HBCx-134 palbo-R31). Statistical significance was calculated using unpaired *t*-test. Data was available for four to seven animals per group. *p < 0.05; **p < 0.01; ***p < 0.001. Error bars represent means ± SEM.

To validate the results obtained in our in vitro experiments, we tested the anti-tumour activity of AZD1775, neratinib or everolimus in the presence or absence of Fulv in an in vivo PDX model of metastatic ER + CCND1-driven BC with acquired resistance to palbociclib (HBCx-134 palbo-R31) (Fig. 6). Palbociclib was maintained in all treatment arms. After 60 days of treatment, neratinib alone or in combination with Fulv arrested tumour growth with TGI of 92% and 94%, respectively (*p* < 0.0001). Treatment by AZD1775 inhibited tumour growth with a TGI of 70% (*p* = 0.0025) and 54% when combined to Fulv (*p* = 0.046). Everolimus alone or in combination with Fulv inhibited tumour growth with TGI of 75% (*p* = 0.0006) and 78% (*p* = 0.0004), respectively.

Taken together, these data highlight the potential of targeting upstream growth factor signalling receptors, downstream signalling pathways such as PI3K/mTORC and G2/M associated checkpoint proteins such as WEE1 in palbociclib resistant tumours.

## Discussion

Deregulation of cell cycle leading to uncontrolled proliferation is a hallmark of cancer [31]. The cell cycle is tightly controlled by interplay between cyclins, CDKs and their respective inhibitors. Direct targeting of cell cycle with CDK inhibitors has provided an attractive proposition but until recently few have shown specificity and associated clinical toxicities have been unacceptable [32]. The CDK4/6-RB axis is critical for cell cycle entry and not surprisingly most cancers subvert this axis to promote proliferation; for instance, 19% of BC show amplification of *CDK4* and *CCND1* amplification is associated with endocrine resistance (reviewed by [33]). Recently, inhibitors such as palbociclib, ribociclib and abemaciclib [34] that target CDK4/6 have shown significant clinical efficacy in metastatic ER + BC by prolonging progression-free survival when given in combination with either the AI letrozole as first-line therapy in endocrine sensitive BC [11, 12], or with the selective ER downregulator Fulv, as second-line therapy in endocrine resistant disease [13]. While this represents a major improvement in clinical outcome, patients still relapse with disease that is resistant to CDK4/6 inhibitors, and the pressing clinical question has become whether the disease is then still endocrine responsive post CDK4/6 inhibitors and/or what therapies would then be appropriate to use thereafter.

In this study, we sought to answer some of these issues by addressing the mechanisms involved in the development of resistance to CDK4/6 inhibition. We initially showed that CDK4/6 inhibition in combination with endocrine therapy targets de novo ligand-independent proliferation providing further justification for the use of these agents to target intrinsic endocrine resistance earlier. Indeed, this notion is supported by the recent NeoPalAna study, which showed patients with Ki67 > 10% gained benefit from the combination of an AI and palbociclib, resulting in complete cell cycle arrest (Ki67 < 2.7%) [35]. In addition, our previous studies suggest that the use of an ‘on-treatment’ *E2F* signature, prior to surgery may provide utility in identifying those patients most likely to benefit from such combinations [36, 37] and may conversely, be useful at identifying patients likely to relapse on CDK4/6 inhibitors later in their course of treatment, discussed in more detail below.

We showed that few genetic changes other than loss of *RB1* CN were associated with resistance to palbociclib in ER + BC models and this appeared restricted to one isogenic cell line. Furthermore, the genetic make-up of the parental cell lines did not inform on the mechanism of resistance to CDK4/6 inhibition. This observation is in keeping with our previous studies [25], in which a PDX model partially resistant to ribociclib after long-term exposure to the drug showed expansion of a pre-existing *RB-null* population. The parental tumour, which was isolated from a patient previously treated with endocrine therapy harbouring an *ESR1*^*Y537S*^, *TP53* mutation and loss of genes encoding p16^INK4A^, p15^INK4B^ and p14^ARF^, was shown to be initially sensitive to palbociclib treatment suggesting that these genetic alterations do not impact on response to CDK4/6 perturbation and that long-term exposure led to the expansion of the pre-existing RB-null sub-population. In our current study, despite the primary tumour from which our palbociclib resistant PDX was derived harbouring a *TP53* mutation, the resultant CDK4/6 resistant tumour maintained RB1 expression. This suggests, loss of *TP53* does not influences RB1 expression and does not determine sensitivity to CDK4/6 inhibition.

Clinical studies from PALOMA-3 indicate that loss of RB1 is a rare event, occurring in only 4.7% of patients, whilst *TP53* mutations were shown to be maintained from day one. In conclusion, the genetic make-up of the primary tumour appears insufficient to determine sensitivity or resistance to CDK4/6 inhibition [38].

Further interrogation using transcriptomic and phosphoproteomic profiling showed cell lines retaining RB used kinase re-wiring to circumvent perturbation of CDK4/6. Noteworthy, an increase in growth factor signalling and downstream activation of MAPK was evident, pathways previously associated with resistance to endocrine therapy. As preceding studies have highlighted the ability of altered MAPK [27] and AKT [39] to impact on the ER-cistrome, we interrogated the ER-genomic binding pattern in MCF7 LTED^PalboR^ cells. ChIP-seq analysis showed a loss of ER-binding to ESR1 and FOXA1 motifs and enrichment at SP1 and AP2 motifs, which were concomitant with a decrease in the expression of classical E-regulated genes *PDZK1* and *TFF1* but not *CCND1*. Further interrogation of the transcriptome showed a reduction in dependence on ER for cell cycle progression and a shift towards dependence on SP1 transcription factors, which have been associated with modulation of EGFR expression [40]. Taken together, these data suggested the resistant cell line had undergone long-term adaptation via cell plasticity leading to chromatin remodelling.

In keeping with an earlier report, those CDK4/6 resistant cell lines that maintained ER expression, showed a reduction in sensitivity to Fulv and tamoxifen compared with their respective parental cell lines [41]. However, in contrast to the previous study, no change in *CDK6* CN was evident in our cell line models. We therefore hypothesised that signalling via EGFR/ERBB may provide the dominant mitogenic drive accounting for the enhanced sensitivity to the pan-ERBB inhibitors neratinib and AZD8931. Mechanistically, we showed that blockade of cyclin D1/CDK4 reduced the phosphorylation of tuberous sclerosis 2 (TSC2), relieving the negative feedback loop and increasing expression of EGFR family members [30] which in turn reduced reliance on ER-driven proliferation. Addition of the pan-ERBB inhibitor neratinib decreased phosphorylation of EGFR and ERBB2 and associated with re-expression of E-regulated genes and enhanced sensitivity to Fulv. Blockade of EGFR also caused a reduction in phosphorylation of RET another receptor tyrosine kinase associated with resistance to AI therapy [42]. Previous studies have shown that EGFR mediates activation of RET and that the two receptors may form an activation complex leading to enhanced downstream signalling via MAPK [43].

Taken together these data suggest altered growth factor signalling pathways, which have previously been associated with endocrine resistance appear further enhanced in CDK4/6 resistant cell lines that retain functional RB. This altered signalling network leads to increased expression of *CDK4*, *CCNE1* and *CCNE2*. The Cyclin E1/CDK2 complex inactivates RB by quantum hyper-phosphorylation [44] leading to transcription of the E2F S-phase entry programme and transcription of *CCNA2*. Moreover, increases in cyclin D1 can aid in cyclin E1/CDK2 complex formation by sequestering CDK2 negative regulators p21^CIP1^ and p27^KIP1^ promoting aberrant RB-phosphorylation and cell cycle progression [45]. In turn, enhanced growth factor signalling reduces the requirement for the classical ER-driven transcriptional programme.

Clinical support for this hypothesis comes from the NeoPalAna study, where association between palbociclib resistance and persistent on-treatment expression of E2F targets (*CCND3, CCNE1* and *CDKN2D)* was evident, indicating that continued activation of E2F transcription in resistant tumours, equates to loss of the RB regulon [35].

In order to take a global view of kinases associated with the palbociclib-resistant phenotype, and to identify new therapeutic targets, we carried out siRNA kinome knockdown studies. Not surprisingly, *PIK3CA* loss caused a marked drop in proliferation of all cell lines irrespective of RB status. The mTORC1 inhibitor everolimus and PI3K inhibitor pictisilib suppressed proliferation of all palbociclib-resistant models, suggesting that sequencing of these agents after resistance to CDK4/6 inhibition may provide clinical utility.

Multiple CDKs were highlighted as potential targets including CDK9. However, the CDK9 inhibitor LDC000067 had little impact on cell proliferation. One explanation is that kinases can have both catalytic and structural modalities [46]. CDK9 can regulate transcription by interacting with transcription factors such as STAT3, c-Jun, and B-Myb in a manner that does not depend on catalytic activity [47]. Noteworthy, CDK7, which was upregulated at the transcript level was also identified as a potential therapeutic target. CDK7 has two functions. Firstly, forming a trimer with cyclin H1 and MAT1 to generate the CDK-activating complex, which phosphorylates CDK1, 2, 4 and 6 within the activation segment (T-Loop); and secondly, as a component of the general transcription factor TFIIH, which is involved in transcription initiation and DNA repair, providing the link between transcription and cell cycle [48]. However, comparison of sensitivity to CDK7 inhibition between parental and palbociclib-resistant derivatives showed no alteration with the exception of T47D LTED^PalboR^ cells, which lose expression of both ER and RB. Interestingly, THZ1 has been proposed to be effective in triple negative BC [49]. Nonetheless our data suggests inhibition of CDK7 may prove an effective therapy prior to or after CDK4/6 inhibitor relapse and number of drugs targeting CDK7 are currently under investigation [50, 51].

In summary, the majority of common kinases associated with resistance were connected with growth factor signalling, PI3K/mTOR and G2/M checkpoint including WEE1, which is required for phosphorylation and inactivation of CDK1 and determines cell size prior to mitosis [52]. In confirmation of these observations, the palbociclib-resistant cell lines showed enhanced sensitivity to drugs targeting these signalling axes, and as previously noted [53–56].

Taken together, these data suggest that multiple mechanisms of resistance to CDK4/6 inhibitors can occur leading to deregulation of RB1 regulon either by CN loss, methylation or increased growth factor signalling. We show pathways associated with endocrine resistance can be further upregulated in RB competent yet CDK4/6 resistant cell lines suggesting that some tumours may remain hardwired to the RB/E2F transcriptional axis and use flexibility in kinase signalling to circumvent the G1/S checkpoint by increasing expression of CCNE1 leading to hyperphosphorylation of RB and reducing dependence on ER signalling as a mitogenic driver. This data highlights the high degree of adaptability to CDK4/6 inhibition and the need to screen patients prior to and after relapse on these inhibitors in order to inform on effective drug sequencing.

## Supplementary information

Supplementary Figure S1

Supplementary Figure S2

Supplementary Figure S3

Supplementary Figure S4

Supplementary Figure S5

Supplementary Figure S6

Supplementary Figure S7

Supplementary File S1

Supplementary File S2

Supplementary File S3

Supplementary File S4

## Data Availability

The data supporting the finding from this manuscript have been deposited as follows: data have been deposited with the NCBI gene expression omnibus (GEO) (http://ncbi.nlm.nih.gov/geo/): Gene expression (GSE98987), Exome sequence and ChIP-seq analysis (PRJNA604231). Phosphoproteomics (PRIDE PXD005514) and Proteomics data (PRIDE PXD005611) have been deposited in the ProteomeXchange.

## References

[1] Patani N, Martin LA (2014). Understanding response and resistance to oestrogen deprivation in ER-positive breast cancer. Mol Cell Endocrinol.

[2] Witzel II, Koh LF, Perkins ND (2010). Regulation of cyclin D1 gene expression. Biochem Soc Trans.

[3] Bosco EE, Wang Y, Xu H, Zilfou JT, Knudsen KE, Aronow BJ (2007). The retinoblastoma tumor suppressor modifies the therapeutic response of breast cancer. J Clin Investig.

[4] Dickson C, Fantl V, Gillett C, Brookes S, Bartek J, Smith R (1995). Amplification of chromosome band 11q13 and a role for cyclin D1 in human breast cancer. Cancer Lett.

[5] Gillett C, Fantl V, Smith R, Fisher C, Bartek J, Dickson C (1994). Amplification and overexpression of cyclin D1 in breast cancer detected by immunohistochemical staining. Cancer Res.

[6] Jirstrom K, Stendahl M, Ryden L, Kronblad A, Bendahl PO, Stal O (2005). Adverse effect of adjuvant tamoxifen in premenopausal breast cancer with cyclin D1 gene amplification. Cancer Res.

[7] Rudas M, Lehnert M, Huynh A, Jakesz R, Singer C, Lax S (2008). Cyclin D1 expression in breast cancer patients receiving adjuvant tamoxifen-based therapy. Clin Cancer Res.

[8] Lundgren K, Brown M, Pineda S, Cuzick J, Salter J, Zabaglo L (2012). Effects of cyclin D1 gene amplification and protein expression on time to recurrence in postmenopausal breast cancer patients treated with anastrozole or tamoxifen: a TransATAC study. Breast Cancer Res.

[9] Paternot S, Bockstaele L, Bisteau X, Kooken H, Coulonval K, Roger PP (2010). Rb inactivation in cell cycle and cancer: the puzzle of highly regulated activating phosphorylation of CDK4 versus constitutively active CDK-activating kinase. Cell Cycle.

[10] Fry DW, Harvey PJ, Keller PR, Elliott WL, Meade M, Trachet E (2004). Specific inhibition of cyclin-dependent kinase 4/6 by PD 0332991 and associated antitumor activity in human tumor xenografts. Mol Cancer Ther.

[11] Hortobagyi GN, Stemmer SM, Burris HA, Yap YS, Sonke GS, Paluch-Shimon S (2016). Ribociclib as first-line therapy for HR-positive, advanced breast cancer. N Engl J Med.

[12] Finn RS, Martin M, Rugo HS, Jones S, Im SA, Gelmon K (2016). Palbociclib and letrozole in advanced breast cancer. N Engl J Med.

[13] Turner NC, Ro J, Andre F, Loi S, Verma S, Iwata H (2015). Palbociclib in hormone-receptor-positive advanced breast cancer. N Engl J Med.

[14] Ribas R, Pancholi S, Guest SK, Marangoni E, Gao Q, Thuleau A (2015). AKT antagonist AZD5363 influences estrogen receptor function in endocrine resistant breast cancer and synergises with fulvestrant (ICI182780) in vivo. Mol Cancer Ther.

[15] Martin LA, Ribas R, Simigdala N, Schuster E, Pancholi S, Tenev T (2017). Discovery of naturally occurring ESR1 mutations in breast cancer cell lines modelling endocrine resistance. Nat Commun.

[16] Schmidt D, Wilson MD, Spyrou C, Brown GD, Hadfield J, Odom DT (2009). ChIP-seq: using high-throughput sequencing to discover protein-DNA interactions. Methods.

[17] Stark R, Brown GD. DiffBind: differential binding analysis of ChIP-Seq peak data, 2011.

[18] Simigdala N, Gao Q, Pancholi S, Roberg-Larsen H, Zvelebil M, Ribas R (2016). Cholesterol biosynthesis pathway as a novel mechanism of resistance to estrogen deprivation in estrogen receptor-positive breast cancer. Breast Cancer Res.

[19] Zhou H, Ye M, Dong J, Corradini E, Cristobal A, Heck AJ (2013). Robust phosphoproteome enrichment using monodisperse microsphere-based immobilized titanium (IV) ion affinity chromatography. Nat Protoc.

[20] Ressa A, Fitzpatrick M, Van den Toorn H, Heck AJR, Altelaar M (2018). PaDuA: a Python library for high-throughput (phospho)proteomic data analysis. J. Proteome Res..

[21] Rudolph JD, de Graauw M, van de Water B, Geiger T, Sharan R (2016). Elucidation of signaling pathways from large-scale phosphoproteomic data using protein interaction networks. Cell Syst.

[22] Raaijmakers LM, Giansanti P, Possik PA, Mueller J, Peeper DS, Heck AJ (2015). PhosphoPath: visualization of phosphosite-centric dynamics in temporal molecular networks. J Proteome Res.

[23] Brough R, Frankum JR, Sims D, Mackay A, Mendes-Pereira AM, Bajrami I (2011). Functional viability profiles of breast cancer. Cancer Discov.

[24] Smith EJ, Leone G, Nevins JR (1998). Distinct mechanisms control the accumulation of the Rb-related p107 and p130 proteins during cell growth. Cell Growth Differ.

[25] Herrera-Abreu MT, Palafox M, Asghar U, Rivas MA, Cutts RJ, Garcia-Murillas I (2016). Early adaptation and acquired resistance to CDK4/6 inhibition in estrogen receptor-positive breast cancer. Cancer Res.

[26] Rondon-Lagos M, Verdun Di Cantogno L, Marchio C, Rangel N, Payan-Gomez C, Gugliotta P (2014). Differences and homologies of chromosomal alterations within and between breast cancer cell lines: a clustering analysis. Mol Cytogenet.

[27] Lupien M, Meyer CA, Bailey ST, Eeckhoute J, Cook J, Westerling T (2010). Growth factor stimulation induces a distinct ER(alpha) cistrome underlying breast cancer endocrine resistance.. Genes Dev.

[28] Zacharek SJ, Xiong Y, Shumway SD (2005). Negative regulation of TSC1-TSC2 by mammalian D-type cyclins. Cancer Res.

[29] Chandarlapaty S, Sawai A, Scaltriti M, Rodrik-Outmezguine V, Grbovic-Huezo O, Serra V (2011). AKT inhibition relieves feedback suppression of receptor tyrosine kinase expression and activity. Cancer Cell.

[30] Goel S, Wang Q, Watt AC, Tolaney SM, Dillon DA, Li W (2016). Overcoming therapeutic resistance in HER2-positive breast cancers with CDK4/6 inhibitors. Cancer Cell.

[31] Hanahan D, Weinberg RA (2011). Hallmarks of cancer: the next generation. Cell.

[32] Asghar U, Witkiewicz AK, Turner NC, Knudsen ES (2015). The history and future of targeting cyclin-dependent kinases in cancer therapy. Nat Rev Drug Discov.

[33] Musgrove EA, Caldon CE, Barraclough J, Stone A, Sutherland RL (2011). Cyclin D as a therapeutic target in cancer. Nat Rev Cancer.

[34] Goetz MP, Toi M, Campone M, Sohn J, Paluch-Shimon S, Huober J (2017). MONARCH 3: abemaciclib as initial therapy for advanced breast cancer. J Clin Oncol.

[35] Ma CX, Gao F, Luo J, Northfelt DW, Goetz M, Forero A (2017). NeoPalAna: Neoadjuvant Palbociclib, a cyclin-dependent kinase 4/6 inhibitor, and anastrozole for clinical stage 2 or 3 estrogen receptor-positive breast cancer. Clin Cancer Res..

[36] Miller TW, Balko JM, Fox EM, Ghazoui Z, Dunbier A, Anderson H (2011). ERalpha-dependent E2F transcription can mediate resistance to estrogen deprivation in human breast cancer. Cancer Discov.

[37] Gao Q, Patani N, Dunbier AK, Ghazoui Z, Zvelebil M, Martin LA (2014). Effect of aromatase inhibition on functional gene modules in estrogen receptor-positive breast cancer and their relationship with antiproliferative response. Clin Cancer Res.

[38] O’Leary B, Cutts RJ, Liu Y, Hrebien S, Huang X, Fenwick K (2018). The genetic landscape and clonal evolution of breast cancer resistance to palbociclib plus fulvestrant in the PALOMA-3 trial. Cancer Discov.

[39] Bhat-Nakshatri P, Wang G, Appaiah H, Luktuke N, Carroll JS, Geistlinger TR (2008). AKT alters genome-wide estrogen receptor alpha binding and impacts estrogen signaling in breast cancer. Mol Cell Biol.

[40] Grinstein E, Jundt F, Weinert I, Wernet P, Royer HD (2002). Sp1 as G1 cell cycle phase specific transcription factor in epithelial cells. Oncogene.

[41] Yang C, Li Z, Bhatt T, Dickler M, Giri D, Scaltriti M (2016). Acquired CDK6 amplification promotes breast cancer resistance to CDK4/6 inhibitors and loss of ER signaling and dependence. Oncogene.

[42] Morandi A, Martin LA, Gao Q, Pancholi S, Mackay A, Robertson D (2013). GDNF-RET signaling in ER-positive breast cancers is a key determinant of response and resistance to aromatase inhibitors. Cancer Res.

[43] Bhinge K, Yang L, Terra S, Nasir A, Muppa P, Aubry MC (2017). EGFR mediates activation of RET in lung adenocarcinoma with neuroendocrine differentiation characterized by ASCL1 expression. Oncotarget.

[44] Narasimha AM, Kaulich M, Shapiro GS, Choi YJ, Sicinski P, Dowdy SF (2014). Cyclin D activates the Rb tumor suppressor by mono-phosphorylation. eLife.

[45] Halaban R, Miglarese MR, Smicun Y, Puig S (1998). Melanomas, from the cell cycle point of view (Review). Int J Mol Med.

[46] Shaw AS, Kornev AP, Hu J, Ahuja LG, Taylor SS (2014). Kinases and pseudokinases: lessons from RAF. Mol Cell Biol.

[47] De Falco G, Bagella L, Claudio PP, De Luca A, Fu Y, Calabretta B (2000). Physical interaction between CDK9 and B-Myb results in suppression of B-Myb gene autoregulation. Oncogene.

[48] Fisher RP (2005). Secrets of a double agent: CDK7 in cell-cycle control and transcription. J Cell Sci.

[49] Li B, Ni Chonghaile T, Fan Y, Madden SF, Klinger R, O’Connor AE (2017). Therapeutic rationale to target highly expressed CDK7 conferring poor outcomes in triple-negative breast cancer. Cancer Res.

[50] Kwiatkowski N, Zhang T, Rahl PB, Abraham BJ, Reddy J, Ficarro SB (2014). Targeting transcription regulation in cancer with a covalent CDK7 inhibitor. Nature.

[51] Patel H, Periyasamy M, Sava GP, Bondke A, Slafer BW, Kroll SHB (2018). ICEC0942, an orally bioavailable selective inhibitor of cdk7 for cancer treatment. Mol Cancer Ther.

[52] Matheson CJ, Backos DS, Reigan P (2016). Targeting WEE1 kinase in cancer. Trends Pharmacol Sci.

[53] Michaloglou C, Crafter C, Siersbaek R, Delpuech O, Curwen JO, Carnevalli LS (2018). Combined inhibition of mTOR and CDK4/6 is required for optimal blockade of E2F function and long-term growth inhibition in estrogen receptor-positive breast cancer. Mol Cancer Ther.

[54] Kettner NM, Vijayaraghavan S, Durak MG, Bui T, Kohansal M, Ha MJ (2019). Combined inhibition of STAT3 and DNA repair in palbociclib-resistant ER-positive breast cancer. Clin Cancer Res.

[55] Jansen VM, Bhola NE, Bauer JA, Formisano L, Lee KM, Hutchinson KE (2017). Kinome-wide RNA interference screen reveals a role for PDK1 in acquired resistance to CDK4/6 inhibition in ER-positive breast cancer. Cancer Res.

[56] de Leeuw R, McNair C, Schiewer MJ, Neupane NP, Brand LJ, Augello MA (2018). MAPK reliance via acquired CDK4/6 inhibitor resistance in cancer. Clin Cancer Res.

