# Supplementary figures and images for: Tumour kinome re-wiring governs resistance to palbociclib in oestrogen receptor positive breast cancers, highlighting new therapeutic modalities
Source: Oncogene. 2020 Apr 19;39(25):4781–97. doi: 10.1038/s41388-020-1284-6 (PMC7299844; doi:10.1038/s41388-020-1284-6)

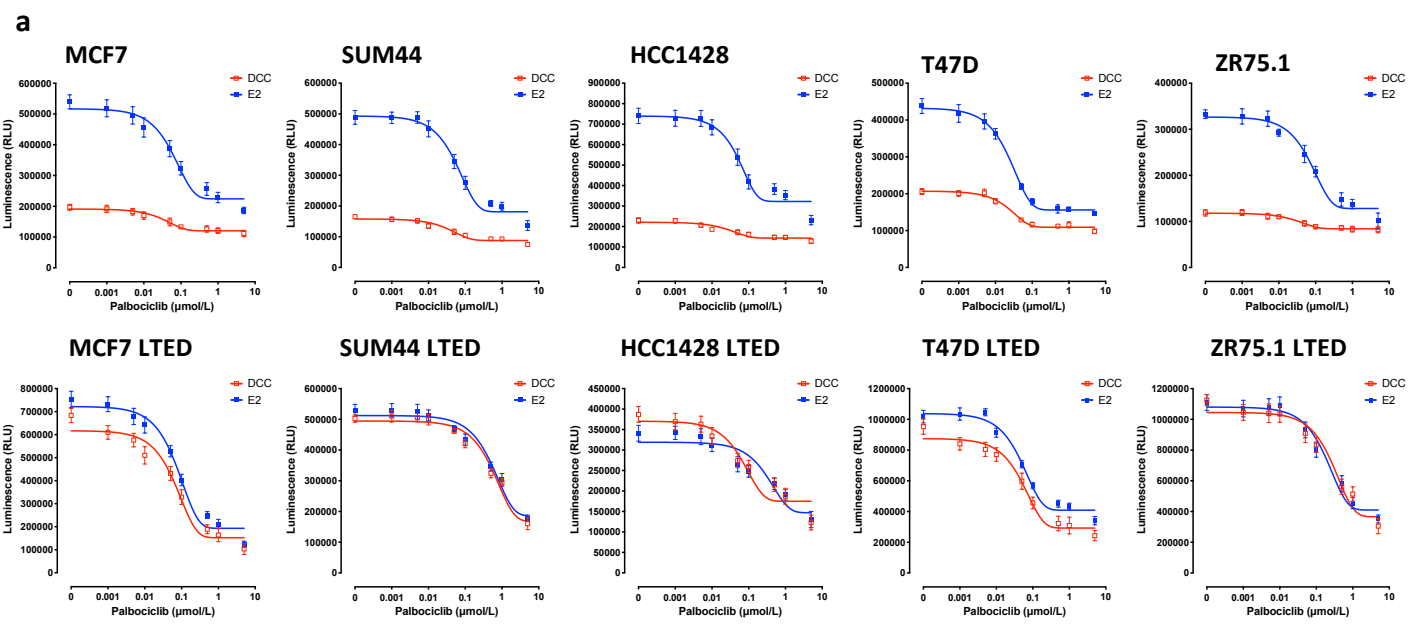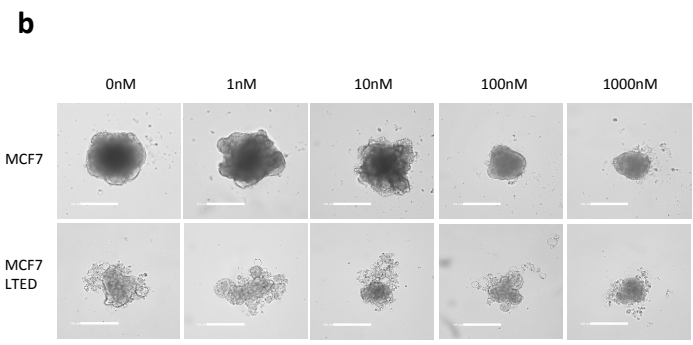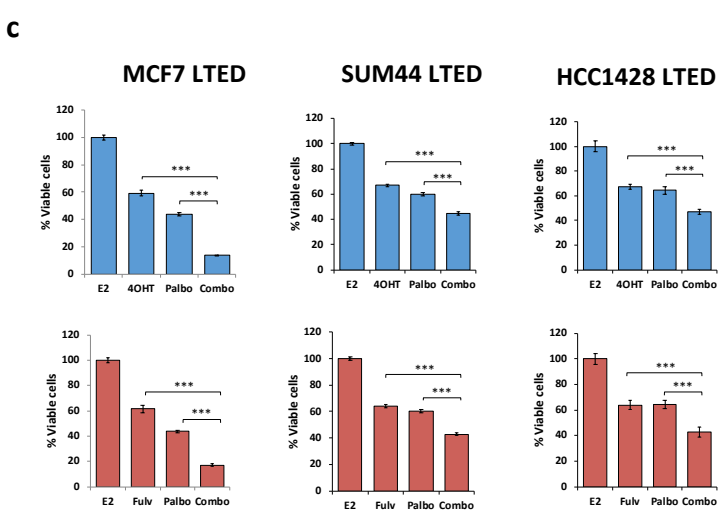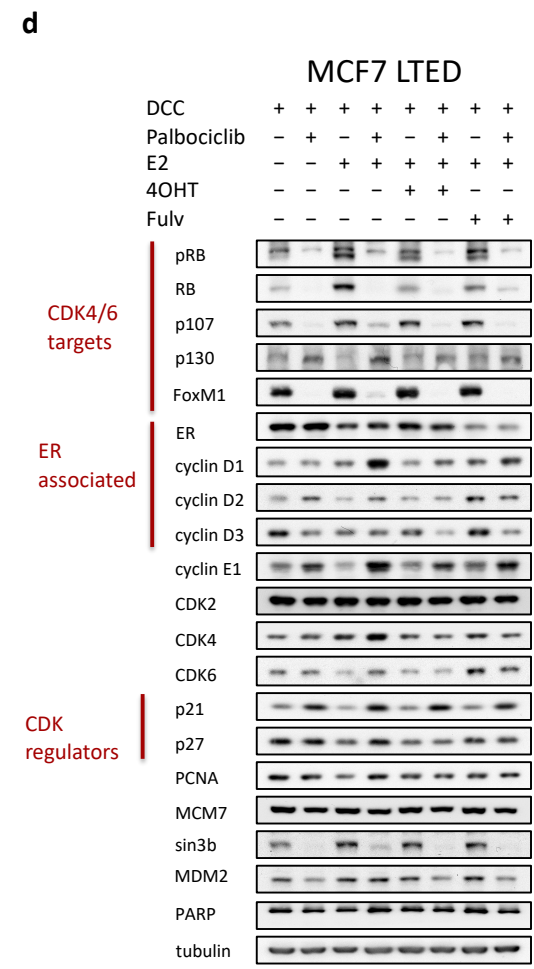

Supplement: Supplementary file 1 — Supplementary Figure S1 [file 41388_2020_1284_MOESM1_ESM.pdf]

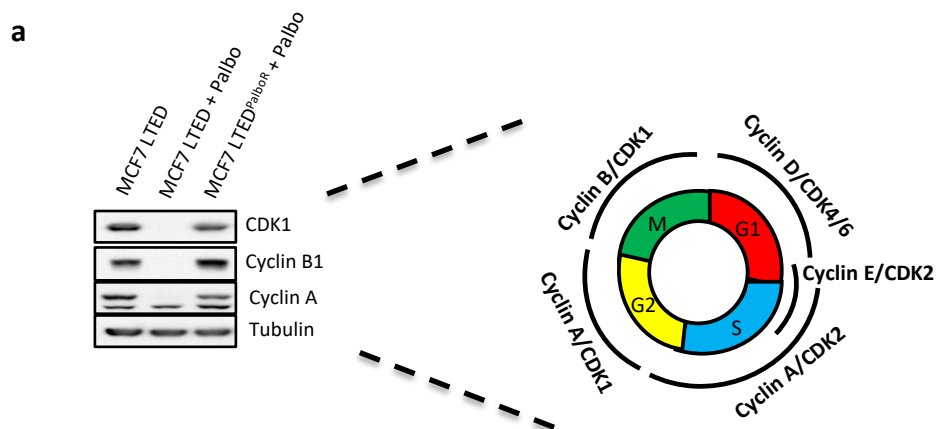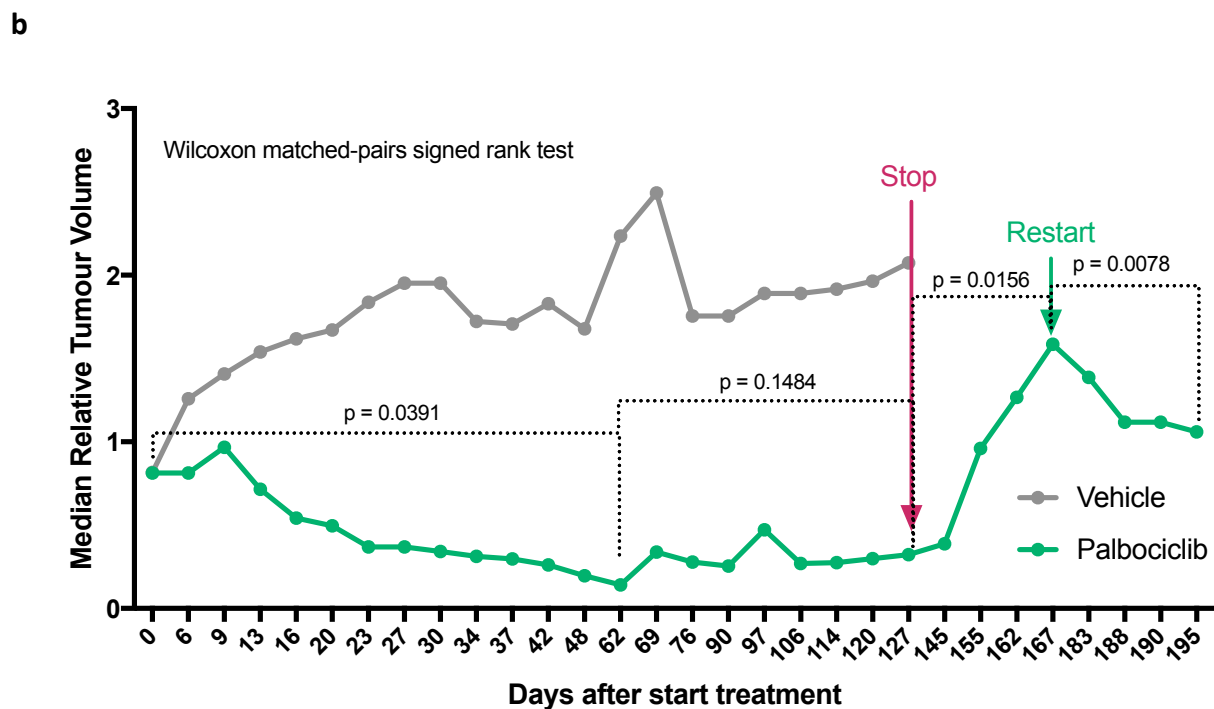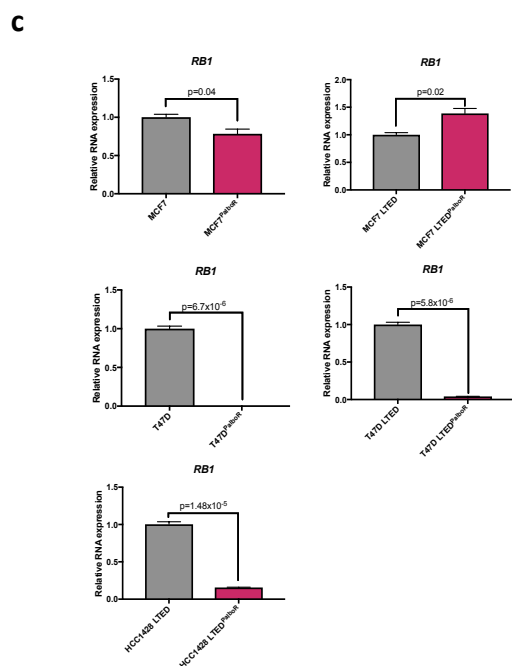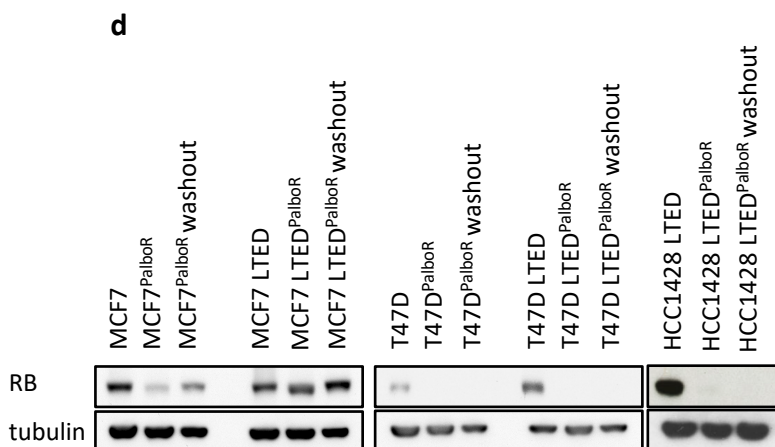

Supplement: Supplementary file 2 — Supplementary Figure S2 [file 41388_2020_1284_MOESM2_ESM.pdf]

a

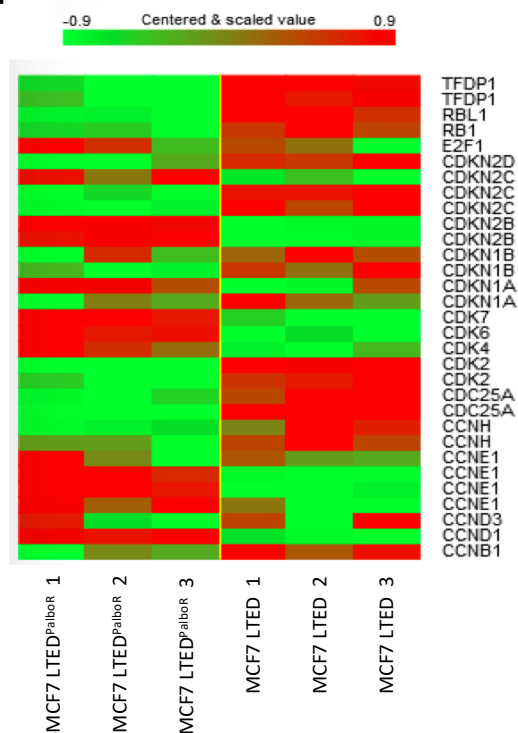

b

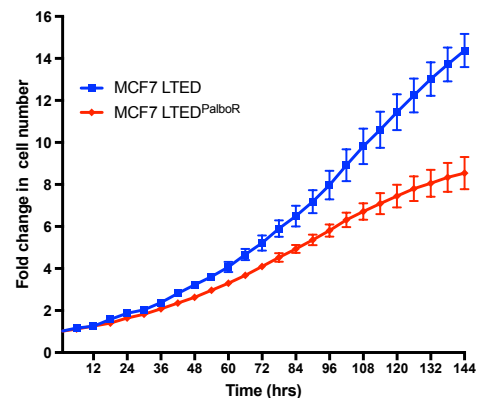

c

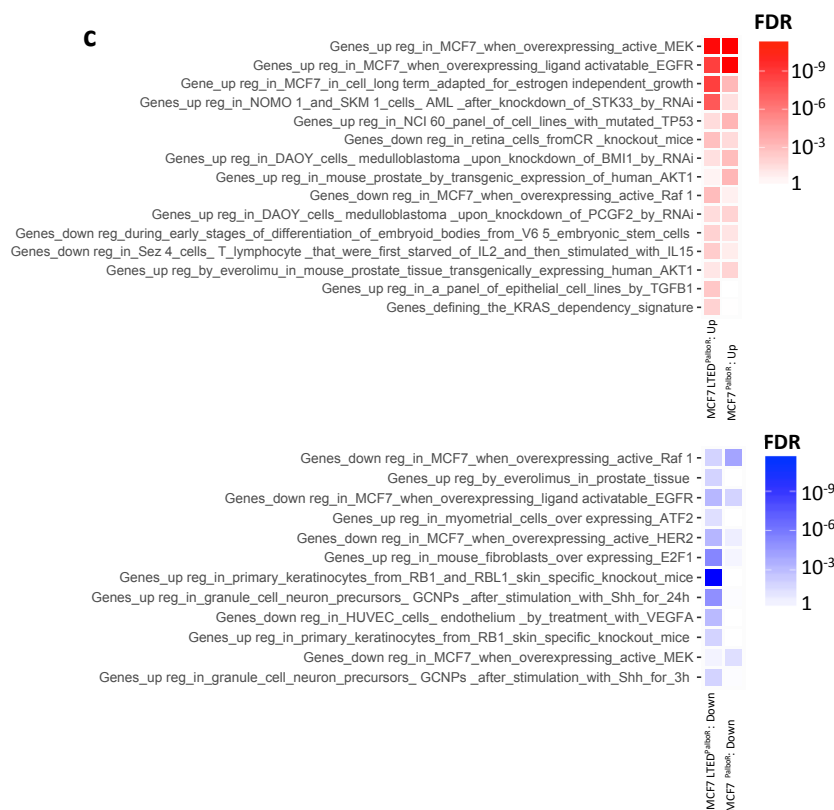

Supplement: Supplementary file 3 — Supplementary Figure S3 [file 41388_2020_1284_MOESM3_ESM.pdf]

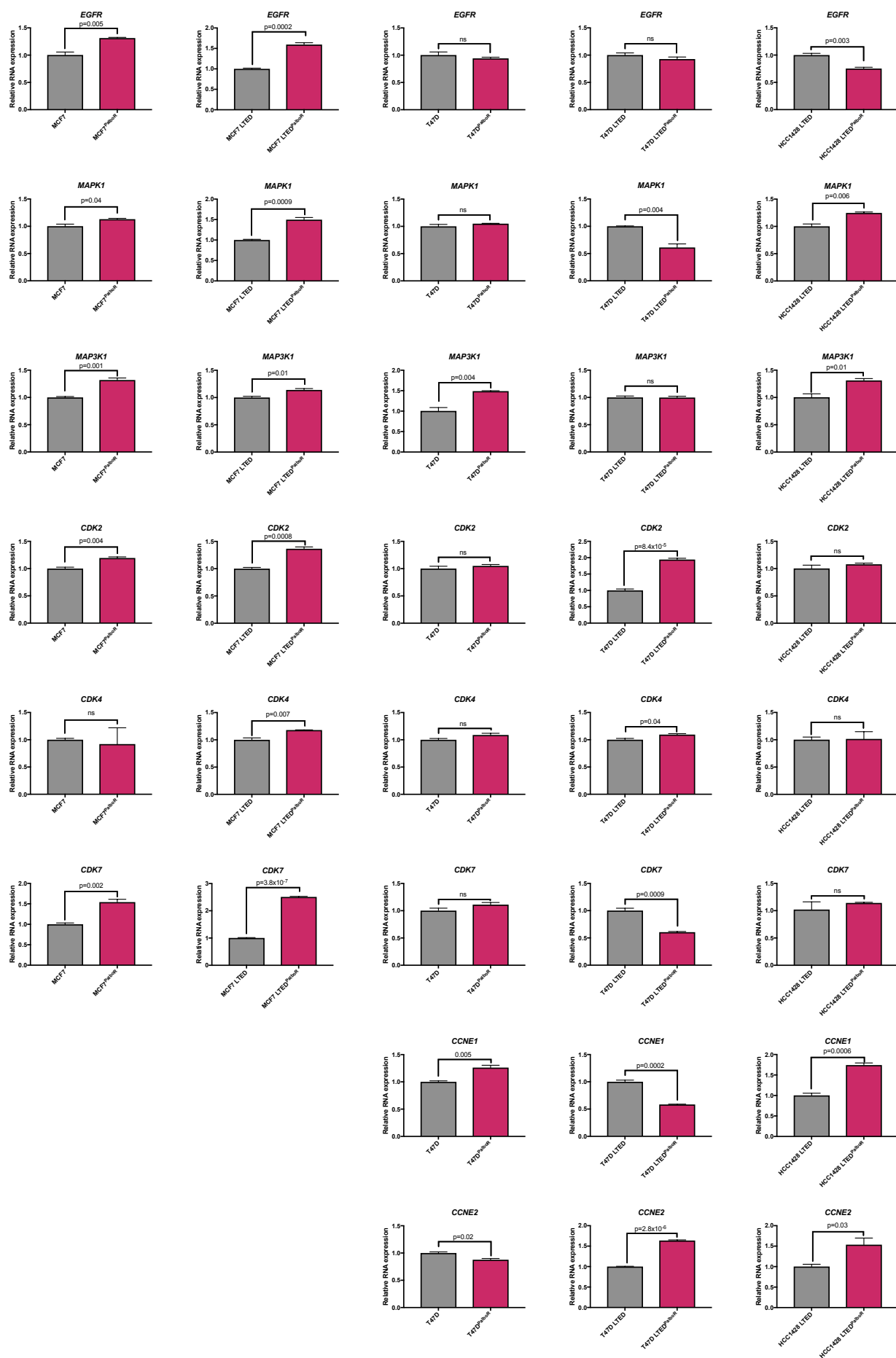

Supplement: Supplementary file 4 — Supplementary Figure S4 [file 41388_2020_1284_MOESM4_ESM.pdf]

**a**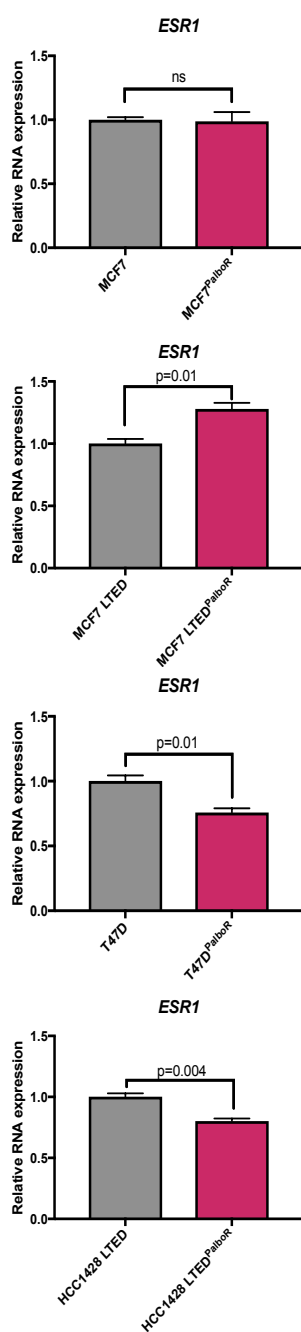**b**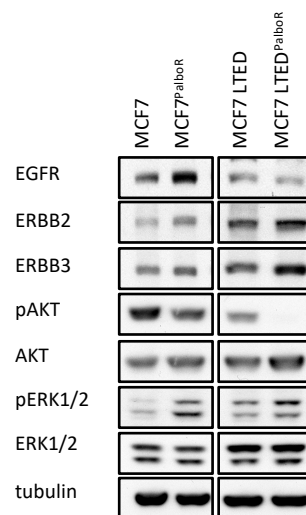

Supplement: Supplementary file 5 — Supplementary Figure S5 [file 41388_2020_1284_MOESM5_ESM.pdf]

a

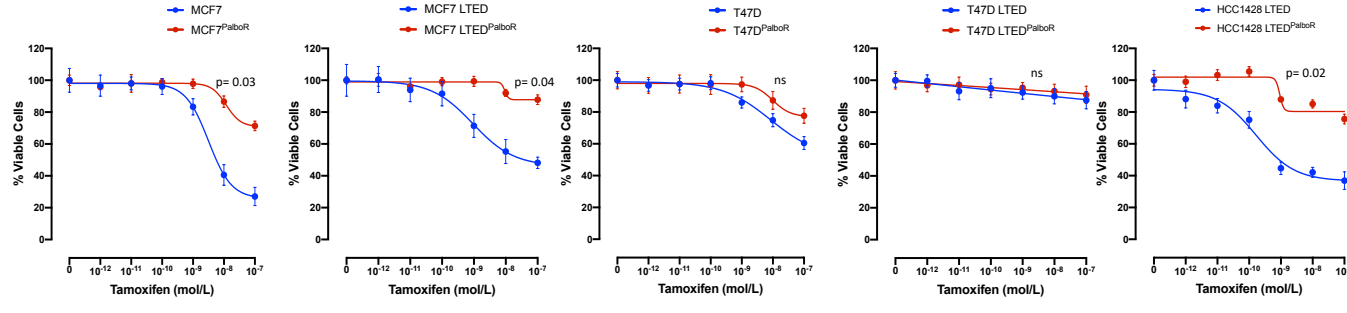

b

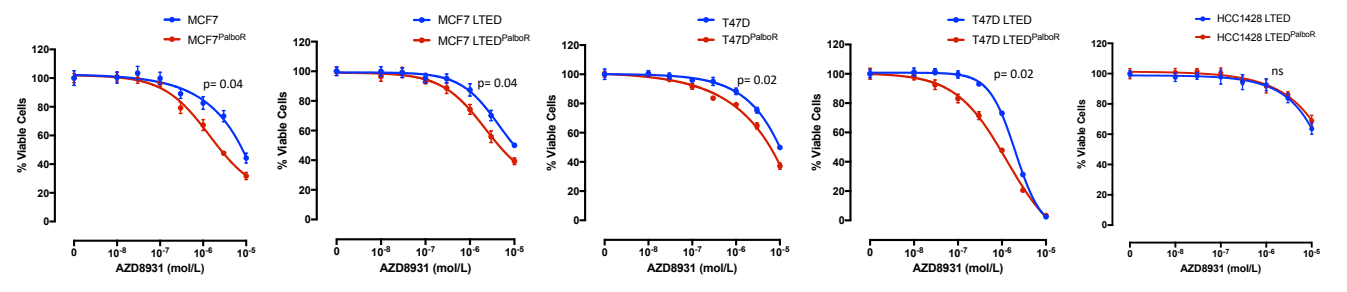

c

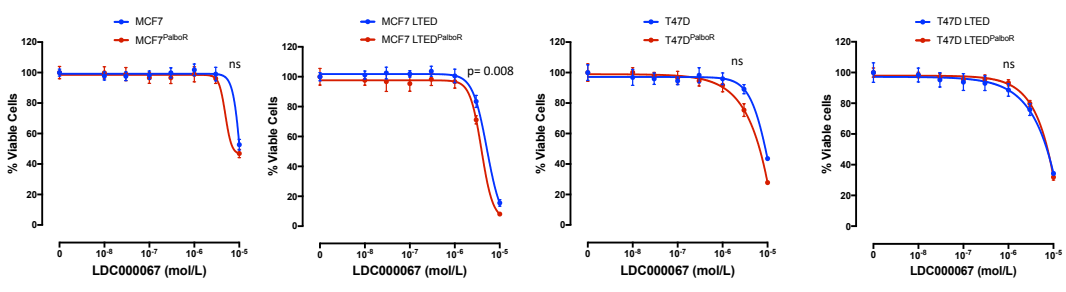

Supplement: Supplementary file 7 — Supplementary Figure S7 [file 41388_2020_1284_MOESM7_ESM.pdf]
