# Supplementary material for: Tumour kinome re-wiring governs resistance to palbociclib in oestrogen receptor positive breast cancers, highlighting new therapeutic modalities
Source: Oncogene. 2020 Apr 19;39(25):4781–97. doi: 10.1038/s41388-020-1284-6 (PMC7299844; doi:10.1038/s41388-020-1284-6)
Supplement: Supplementary file 6 — Supplementary Figure S6 [file 41388_2020_1284_MOESM6_ESM.pdf]

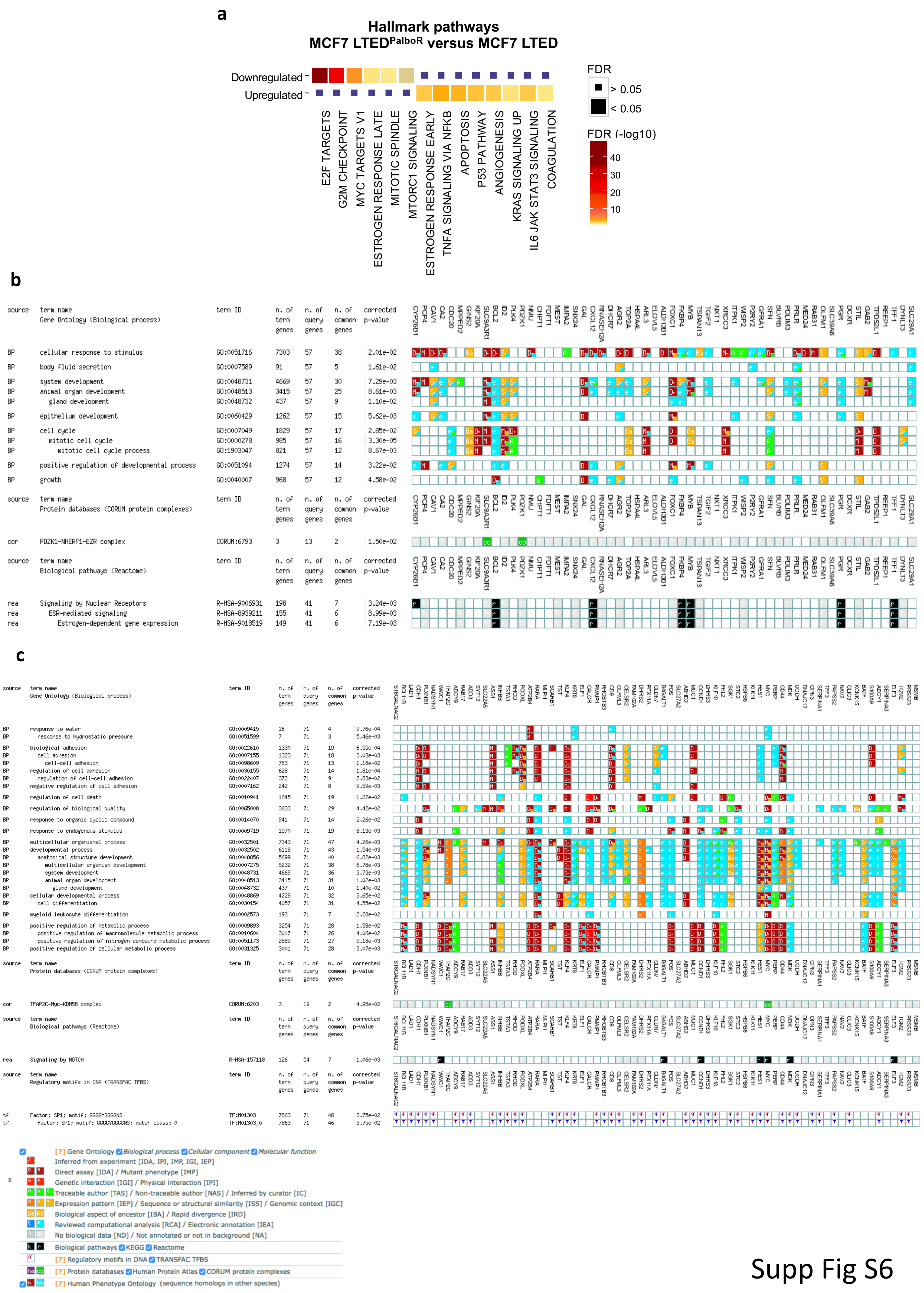
a
Hallmark pathways
MCF7 LTEDPalboR versus MCF7 LTED
Downregulated
Upregulated
E2F TARGETS
G2M CHECKPOINT
MYC TARGETS V1
ESTROGEN RESPONSE LATE
MITOTIC SPINDLE
MTORC1 SIGNALING
ESTROGEN RESPONSE EARLY
TNFA SIGNALING VIA NFKB
APOPTOSIS
P53 PATHWAY
ANGIOGENESIS
KRAS SIGNALING UP
IL6 JAK STAT3 SIGNALING
COAGULATION
FDR
> 0.05
< 0.05
FDR (-log10)
40
30
20
10
b
source term name term ID n. of term genes n. of query genes n. of common genes corrected p-value
Gene Ontology (Biological process)
BP cellular response to stimulus GO:0051716 7303 57 38 2.01e-02
BP body fluid secretion GO:0007589 91 57 5 1.61e-02
BP system development GO:0048731 4669 57 30 7.29e-03
BP animal organ development GO:0048513 3415 57 25 8.61e-03
BP gland development GO:0048732 437 57 9 1.10e-02
BP epithelium development GO:0060429 1262 57 15 5.62e-03
BP cell cycle GO:0007049 1829 57 17 2.85e-02
BP mitotic cell cycle GO:0000278 985 57 16 3.30e-05
BP mitotic cell cycle process GO:1903047 821 57 12 8.67e-03
BP positive regulation of developmental process GO:0051094 1274 57 14 3.22e-02
BP growth GO:0040007 968 57 12 4.58e-02
Protein databases (CORUM protein complexes)
cor PDZK1-NHERF1-EZR complex CORUM:6793 3 13 2 1.50e-02
Biological pathways (Reactome)
rea Signaling by Nuclear Receptors R-HSA-9006931 198 41 7 3.24e-03
rea ESR-mediated signaling R-HSA-8939211 155 41 6 8.99e-03
rea Estrogen-dependent gene expression R-HSA-9018519 149 41 6 7.19e-03
c
Gene Ontology (Biological process)
BP response to water GO:0009415 16 71 4 9.76e-04
BP response to hydrostatic pressure GO:0051599 7 71 3 5.46e-03
BP biological adhesion GO:0022610 1330 71 19 6.55e-04
BP cell adhesion GO:0007155 1323 71 18 3.03e-03
BP cell-cell adhesion GO:0098609 763 71 13 1.18e-02
BP regulation of cell adhesion GO:0030155 628 71 14 1.81e-04
BP regulation of cell-cell adhesion GO:0022407 372 71 9 2.83e-02
BP negative regulation of cell adhesion GO:0007162 242 71 8 9.59e-03
BP regulation of cell death GO:0010941 1645 71 19 1.62e-02
BP regulation of biological quality GO:0065008 3633 71 29 4.42e-02
BP response to organic cyclic compound GO:0014070 941 71 14 2.26e-02
BP response to endogenous stimulus GO:0009719 1570 71 19 8.13e-03
BP multicellular organismal process GO:0032501 7343 71 47 4.26e-03
BP developmental process GO:0032502 6118 71 43 1.54e-03
BP anatomical structure development GO:0048856 5699 71 40 6.82e-03
BP multicellular organism development GO:0007275 5232 71 38 6.78e-03
BP system development GO:0048731 4669 71 36 3.73e-03
BP animal organ development GO:0048513 3415 71 31 1.02e-03
BP gland development GO:0048732 437 71 10 1.40e-02
BP cellular developmental process GO:0048869 4229 71 32 3.65e-02
BP cell differentiation GO:0030154 4057 71 31 4.55e-02
BP myeloid leukocyte differentiation GO:0002573 193 71 7 2.28e-02
BP positive regulation of metabolic process GO:0009893 3254 71 28 1.58e-02
BP positive regulation of macromolecule metabolic process GO:0010604 3017 71 26 4.06e-02
BP positive regulation of nitrogen compound metabolic process GO:0051173 2889 71 27 5.18e-03
BP positive regulation of cellular metabolic process GO:0031325 3001 71 28 3.07e-03
Protein databases (CORUM protein complexes)
cor TFAP2C-Myc-KDM5B complex CORUM:6203 3 19 2 4.95e-02
Biological pathways (Reactome)
rea Signaling by NOTCH R-HSA-157118 126 54 7 1.06e-03
Regulatory motifs in DNA (TRANSFAC TFBS)
tf Factor: SP1; motif: GGGGYGGGGNS TF:M01303 7863 71 46 3.75e-02
tf Factor: SP1; motif: GGGGYGGGGNS; match class: 0 TF:M01303_0 7863 71 46 3.75e-02
[?] Gene Ontology Biological process Cellular component Molecular function
Inferred from experiment [IDA, IPI, IMP, IGI, IEP]
Direct assay [IDA] / Mutant phenotype [IMP]
Genetic interaction [IGI] / Physical interaction [IPI]
Traceable author [TAS] / Non-traceable author [NAS] / Inferred by curator [IC]
Expression pattern [IEP] / Sequence or structural similarity [ISS] / Genomic context [IGC]
Biological aspect of ancestor [IBA] / Rapid divergence [IRD]
Reviewed computational analysis [RCA] / Electronic annotation [IEA]
No biological data [ND] / Not annotated or not in background [NA]
Biological pathways KEGG Reactome
[?] Regulatory motifs in DNA TRANSFAC TFBS
[?] Protein databases Human Protein Atlas CORUM protein complexes
[?] Human Phenotype Ontology (sequence homologs in other species)

Supp Fig S6
